# Δ^9^-Tetrahydrocannabinol Alters Limbic and Frontal Functional Brain Connectomes Among Young Adult Cannabis Users

**DOI:** 10.1016/j.bpsc.2025.09.005

**Published:** 2025-09-14

**Authors:** Zachary Anderson, Matthew Gunn, Emily Jones, Olusola Ajilore, K. Luan Phan, Harriet de Wit, Heide Klumpp, Vince Calhoun, Natania A. Crane

**Affiliations:** From the Department of Psychology, Northwestern University, Evanston, Illinois (ZA); Department of Psychiatry, University of Illinois Chicago, Chicago, Illinois (ZA, MG, EJ, OA, HK, NAC); Department of Psychiatry and Behavioral Health, Wexner Medical Center, Ohio State University, Columbus, Ohio (KLP); Department of Psychiatry and Behavioral Neuroscience, University of Chicago, Chicago, Illinois (HdW); Psychology Department and Neuroscience Institute, Georgia State University, Atlanta, Georgia (VC); and Tri-Institutional Center for Translational Research in Neuroimaging and Data Science, Georgia State University, Georgia Institute of Technology and Emory University, Atlanta, Georgia (VC).

## Abstract

**BACKGROUND:**

Cannabis use among young adults has reached the highest levels ever recorded. Evidence indicates that acute Δ^9^-tetrahydrocannabinol (THC) disrupts brain connectivity. Few studies have examined this on a whole-brain level. We examined the effects of a single moderate dose of THC on resting-state functional brain networks among young adult cannabis users.

**METHODS:**

In a within-subject, double-blind, randomized study, 33 healthy occasional cannabis users received THC (7.5 mg, oral) and placebo before completing resting-state functional magnetic resonance imaging (rs-fMRI) during peak intoxication. Group-information–guided independent component analysis was performed on resting-state brain data to identify whole-brain networks associated with each scan. Within-samples *t* tests assessed for differences in intrinsic network functional connectivity and between-network functional connectivity after THC versus placebo. Additional linear models examined relationships between brain connectivity, subjective drug effects, and past-month cannabis use.

**RESULTS:**

THC reduced within-network intrinsic connectivity in corticostriatal circuits and other networks associated with sensory systems, interoceptive experiences, and spatial reasoning. THC reduced connectivity between 2 networks characterized by the anterior cingulate cortex and dorsal insula regions as well as the ventral insula and lingual gyrus, respectively. Network connectivity during THC (vs. placebo) was not related to subjective measures of drug effect or recent cannabis use.

**CONCLUSIONS:**

Our findings add to a growing literature showing that THC decreases rs-fMRI throughout the brain, impacting networks linked to the many behavioral and perceptual changes associated with THC. Future work is needed to extend these findings to clinical samples and to assess the extent to which these networks are associated with negative outcomes of chronic THC use.

Cannabis use among young adults has reached historic highs (1), with increases in both prevalence and quantity used (2,3). This coincides with heightened Δ^9^-tetrahydrocannabinol (THC) potency of street cannabis, which has significantly increased over the past 2 decades (4-6). THC is the primary psychoactive component in cannabis, linked to the addictive, rewarding properties of cannabis (7). Use of high-THC-potency cannabis is related to elevated risk of depression, anxiety, addiction, and psychosis (8-14). THC binds to the endogenous cannabinoid system, which increases dopamine transmission in the cortico-striato-limbic system (15-24). Accumulating evidence indicates that acute THC disrupts brain connectivity at the circuit level (25), but few studies have examined whole-brain effects or interactions between interconnected networks that are directly related to a number of cognitive and emotion processes (26). Therefore, we used group-information–guided independent component analysis (GIG-ICA) on resting-state functional magnetic resonance imaging (rs-fMRI) data to examine how THC (vs. placebo [PBO]) impacts within- and between-network functional connectivity among young adults who use cannabis.

rs-fMRI allows researchers to measure THC-induced disruptions in functional connectivity across large-scale networks without confounds such as task performance, cognitive demands, or behavioral variability (27). However, the results of previous work examining acute effects of THC on rs-fMRI connectivity remain inconsistent, likely due to methodological differences, individual variability in intoxication response, cannabis use history, and small sample sizes (>75% studies; *N* < 25) (28,29). For example, a general trend suggests that acute THC reduces rs-fMRI connectivity in cortico-striato-limbic circuits, particularly among cannabis users (29,30). In contrast, our group found that noncannabis users (<10 lifetime uses) who received 7.5 mg THC exhibited increased cortico-striato-limbic connectivity compared with PBO (31). Additional evidence suggests that acute THC enhances cortico-striato-limbic connectivity in individuals who use cannabis <1× per week (32-34). Results from these studies suggest that prior cannabis exposure may influence the direction of THC’s effects on large-scale brain networks and provide possible indicators of acute THC effects.

Previous evidence has related THC-induced alterations in brain connectivity to the drug’s subjective effects, but the relationships are inconsistent. In one study, greater subjective intoxication was associated with reduced rs-fMRI connectivity between the ventral striatum and the prefrontal cortex (30) as well as the posterior cingulate cortex (35). In another study, greater subjective intoxication was associated with reduced connectivity within the dorsal attention, limbic, and subcortical networks (23). Altered perceptions after THC are associated with reduced rs-fMRI connectivity between subcortical regions such as the caudate nucleus and sensory processing networks (36). Another study reported that greater subjective reward after THC in nonusers was associated with greater nucleus accumbens–dorsomedial prefrontal cortex connectivity (31). Taken together, the current evidence suggests that THC-induced alterations in cortico-striato-limbic connectivity are related to subjective experiences, but the direction of these effects varies by study. More work is needed to understand this relationship.

One method used to measure functional connectivity is GIG-ICA. GIG-ICA refers to group ICA followed by single-participant spatially constrained ICA for back reconstruction (37), which enhances analytic sensitivity to group differences in neuroimaging studies (38,39). Previous work suggests that this method effectively removes physiological noise, particularly in rs-fMRI data (37). GIG-ICA methods have been reviewed in depth elsewhere (38,40), and corresponding individual-specific spatial and temporal components that describe whole-brain functional connectivity have been applied in several studies (41). Here, we applied GIG-ICA to understand THC-induced effects on rs-fMRI connectivity. We believe that our work sheds new light on functional brain network dynamics and provides methodological and conceptual innovations.

In this study, we characterized effects of acute THC on rs-fMRI brain networks among abstinent young adult cannabis users. Using a within-subject design, we compared individual-specific GIG-ICA rs-fMRI networks collected after THC and PBO. We made several predictions. As in previous work (29), we expected that THC would reduce intrinsic connectivity in distributed brain networks including the reward, cognitive control, salience, and default mode networks. Consistent with previous reports, we expected that acute THC would alter striatal connectivity with the ventromedial prefrontal cortex and anterior cingulate cortex (29,31). In addition, using ratings of “feeling high” in relation to GIG-ICA networks, we hypothesized that subjective experiences would align with alterations across distributed brain networks (23,31).

## METHODS AND MATERIALS

### Participants

Participants were recruited from the community as part of a registered clinical trial (NCT04512365) via advertisements posted throughout the Chicago metropolitan area (e.g., college campuses, local community centers), social media advertisements, and word-of-mouth referrals. Participants provided written informed consent prior to study participation. Study procedures were conducted at the University of Illinois at Chicago (UIC) and were approved by the university’s Institutional Review Board. Participants were compensated for their time.

Participants were required to be 18 to 25 years of age, be able to give informed consent, be fluent in English, report using cannabis at least 10 times in their life (but less than daily), and have a body mass index in the range of 18 to 30. We recruited this population to better understand how acute THC impacts neural reward circuitry in healthy young adults before they develop serious forms of psychopathology. Future work can expand on these findings in clinical samples. Exclusion criteria included <12 years of education; current night shift worker; current or lifetime DSM-5 diagnosis of psychosis, mania, attention-deficit/hyperactivity disorder, obsessive-compulsive disorder, feeding and eating disorder, posttraumatic stress disorder, substance use disorder (SUD) (except for mild or moderate cannabis use disorder or alcohol use disorder); significant depression or anxiety symptoms (>7 on Hamilton Depression or Anxiety Rating Scales); alcohol use >4 days/week; >20 cigarettes/week (or electronic nicotine delivery system equivalent); desire to cut down/stop cannabis use, current engagement in SUD treatment; use of psychoactive medications during the past 4 weeks; cognitive dysfunction (e.g., history of head injury with >5-minute loss of consciousness, intellectual disability, organic mental/neurological syndrome, pervasive developmental disorder); or MRI contraindications (current pregnancy, left-handedness, presence of ferrous-containing metal in body, claustrophobia). Additionally, participants who exceeded an average framewise displacement of 0.3 mm during rs-fMRI scanning were excluded from the analytic sample (*n* = 3). Following exclusions, 33 individuals were included in the final sample. Demographic information for these individuals is presented in Table 1.

### Study Procedure

Interested individuals completed a brief survey and were contacted via telephone to assess their eligibility. Eligible participants completed a screening visit, during which informed consent was obtained and cannabis use history was determined. For blinding purposes during the informed consent procedure, participants were informed that they might receive a stimulant, sedative, cannabinoid, or PBO. Qualifying participants completed a within-subject, double-blind, randomized study using a crossover design in which they attended 2 drug administration visits, approximately 4 to 7 days apart on which they received oral 7.5 mg THC (dronabinol) or PBO, 120 minutes prior to completing fMRI, corresponding with the expected peak plasma and subjective effects of THC. Participants were asked to abstain from all substance use for at least 24 hours before each study session, which was verified by breath and urine samples (although positive THC test results were allowed given the long half-life of THC). Individuals reported on the extent to which they had used THC during the past month. One individual reported cannabis use hours before one of their drug administration sessions. Several others reported using cannabis more than 24 hours prior (mean = 25.69 days).

### Subjective Drug Response

Participants completed standardized measures of subjective drug effects, including the Drug Effects Questionnaire (DEQ) (42) and the Addiction Research Center Inventory (ARCI) (43) at baseline (0 minutes; prior to drug administration) and 30, 60, 90, and 120 minutes (pre-fMRI scanning); 180 minutes (post-fMRI scan); and 240 and 300 minutes (end of session). On the DEQ, participants rated their responses to the questions, “Do you feel any drug effects?” and “Do you feel high right now?” (rated from “not at all” = 0 to “very strongly” = 100). The ARCI includes the morphine-benzedrine group (MBG) (euphoric effects) subscale, which represents the positive, rewarding effects of drugs (44). Peak change difference scores were collected across subjective drug response measures and used to assess the extent to which subjective drug response was related to fMRI measures (45).

### fMRI Acquisition

Participants completed a T1-weighted structural scan and an 8-minute eyes-open rs-fMRI scan in a 3T GE MRI scanner at the UIC Center for Magnetic Resonance Research. Participants fixated on a cross in the middle of the screen and were instructed to let their minds wander. Functional images were acquired using gradient-echo echo-planar images (TR = 2 seconds, TE = 25 ms, flip angle = 82°, 64 × 64 matrix, FOV = 200 mm, slice thickness = 3 mm, with 44 axial slices).

### fMRI Preprocessing

Preprocessing was performed using fMRIPrep version 23.2.1 (46,47), which is based on Nipype version 1.8.6 (48,49). This pipeline applies standard preprocessing steps to brain imaging data. Specific components of this pipeline are described in the Supplement, Section S1, and can also be referenced online (https://fmriprep.org/en/20.2.6/workflows.html).

### GIG-ICA Algorithm

GIG-ICA is effectively group ICA using spatially constrained ICA as the back reconstruction and is described extensively elsewhere (38,39,50). In brief, GIG-ICA first implemented a principal component analysis with the group data to reduce dimensionality at the participant level. Reduced participant data were then concatenated and input into a second principal component analysis, further reducing data dimensionality at the group level. ICA was used to extract information from these reduced data, which provided group-level components. The single-participant components could then be estimated using back reconstruction. In the case of GIG-ICA, we performed spatially constrained ICA (i.e., ICA with reference) with the group maps provided as spatial priors/templates for each participant. This generated a series of individual-specific networks that corresponded with group components but contained individual-specific information. This optimized the independence of fMRI data while maintaining good spatial correspondence between individuals to ease group-level comparisons.

Following GIG-ICA, group-level components were visually inspected to determine whether they represented signal or noise. This was performed by a trained fMRI technician using validated methods (51). The number of clusters, the overlap with gray matter, the size of relevant clusters, and the location relative to regions where signal loss is often present guided exclusionary decisions. With respect to spectral power, increased high-frequency power is generally thought to reflect noise (52), while at least one strong peak between 0.01 and 0.1 Hz is generally consistent with signal. In this way, we identified noise components that likely characterize physiological or motion-related noise in the fMRI data (41). These noise components (components 1, 4, 9–11, 15, 19, 22, 24, 27) were removed from subsequent analyses.

We used an internal GIFT function (50) to label each component that passed the quality check (icatb_componentLabeller). Naming conventions come from the Neuromark 2.0 atlas (53), which is informed by known rs-fMRI networks (53-56). This atlas has been used in a growing body of work (53,54,57-59), and while it is specialized for ICA-related components, it is intended to address issues with replicability in neuroimaging work (60). Networks include a primary domain (i.e., visual domain [VI], paralimbic domain [PL], cerebellar domain) and can include a subdomain (i.e., central executive subdomain, default mode subdomain [DM]). For a full description of how this atlas was generated and the brain regions associated with each network, see work by Jensen *et al.* (53).

### Analysis Plan

In the current work, we were interested in 2 primary characteristics of rs-fMRI functional brain networks: 1) intrinsic connectivity within functional brain networks and 2) functional network connectivity. We performed within-subject analysis on these measures of rs-fMRI connectivity to relate brain changes after THC versus PBO. We also performed tests to relate specific subjective effects of THC to altered rs-fMRI.

Emerging findings suggest that intrinsic activity within functional brain networks are related to underlying patterns of blood oxygen level–dependent (BOLD) signal fluctuations across different frequency bands. Recent work identified 2 low-frequency bands (0.01–0.1 Hz and 0.12–0.25 Hz) that characterize intrinsic connectivity (61). Alterations in these frequency bands are related to a variety of clinical disorders (40,61), highlighting the utility of approaches that decompose BOLD signal into its constituent parts. Therefore, our first outcome of interest was the presence of significant differences in the presence of spectral power across different frequency bands. We performed a series of paired-sample *t* tests using a within-subject design to assess the relationship between intrinsic connectivity and THC intoxication. Then, we performed a second set of analyses that assessed the relationship between subjective THC effects (DEQ high and feel and ARCI-MBG) and intrinsic connectivity. Analyses adjusted for age, sex, average framewise displacement during the PBO and THC scans, and average blood pressure measured immediately before and after the scan session. Analyses were run with an uncorrected threshold (*p* < .001) and a false discovery rate (FDR)–corrected threshold (*p*_FDR_ < .05).

We were also interested in how different functional networks might relate to one another. Interconnections between networks form complex graphical structures that have important implications for brain function (62). Greater understanding of how THC impacts whole-brain rs-fMRI functional brain networks is important. We performed a series of paired-sample *t* tests comparing the THC scan with PBO scan to address this question. Then, we performed a second set of analyses that assessed the relationship between subjective THC effects (DEQ high and feel and ARCI-MBG) and functional network connectivity. Because there is little evidence from previous studies about the effects of THC on network rs-fMRI dynamics, we did not make specific predictions regarding this measure and reported all findings with respective statistical thresholds. Prior to analysis, a default bandpass filter was applied to component data (0.1 < Hz < 0.15) (52). All analyses adjusted for age, sex, average framewise displacement during the PBO and THC scans, and average blood pressure measured immediately before and after the scan session. In the case of significant findings, we applied additional covariates to rule out the potential impact of other substances such as nicotine and alcohol. Two separate regressors were entered into our models to adjust for the number of days since an individual had used either nicotine or alcohol. Two additional regressors were included to adjust for the total number of alcoholic beverages and nicotine products that were consumed. Analyses were run with an uncorrected threshold (*p* < .001) as well as an FDR-corrected threshold (*p*_FDR_ < .05).

## RESULTS

Thirty components were initially generated in the current work (see the Supplement, Section S2). Of these components, 20 were deemed appropriate for analysis (components 2–3, 5–8, 12–14, 16–18, 20–21, 23, 25–26, 28–30), because they displayed a reasonable power spectrum diagram with a single spike in frequency and did not contain known visual patterns (e.g., ringing) that typically indicate that a component is too noisy for use. Components also aligned with known brain regions and only minimally included cerebrospinal fluid or white matter. These 20 components were entered into the remaining analyses.

### Subjective Drug Response

**Figure 1.**
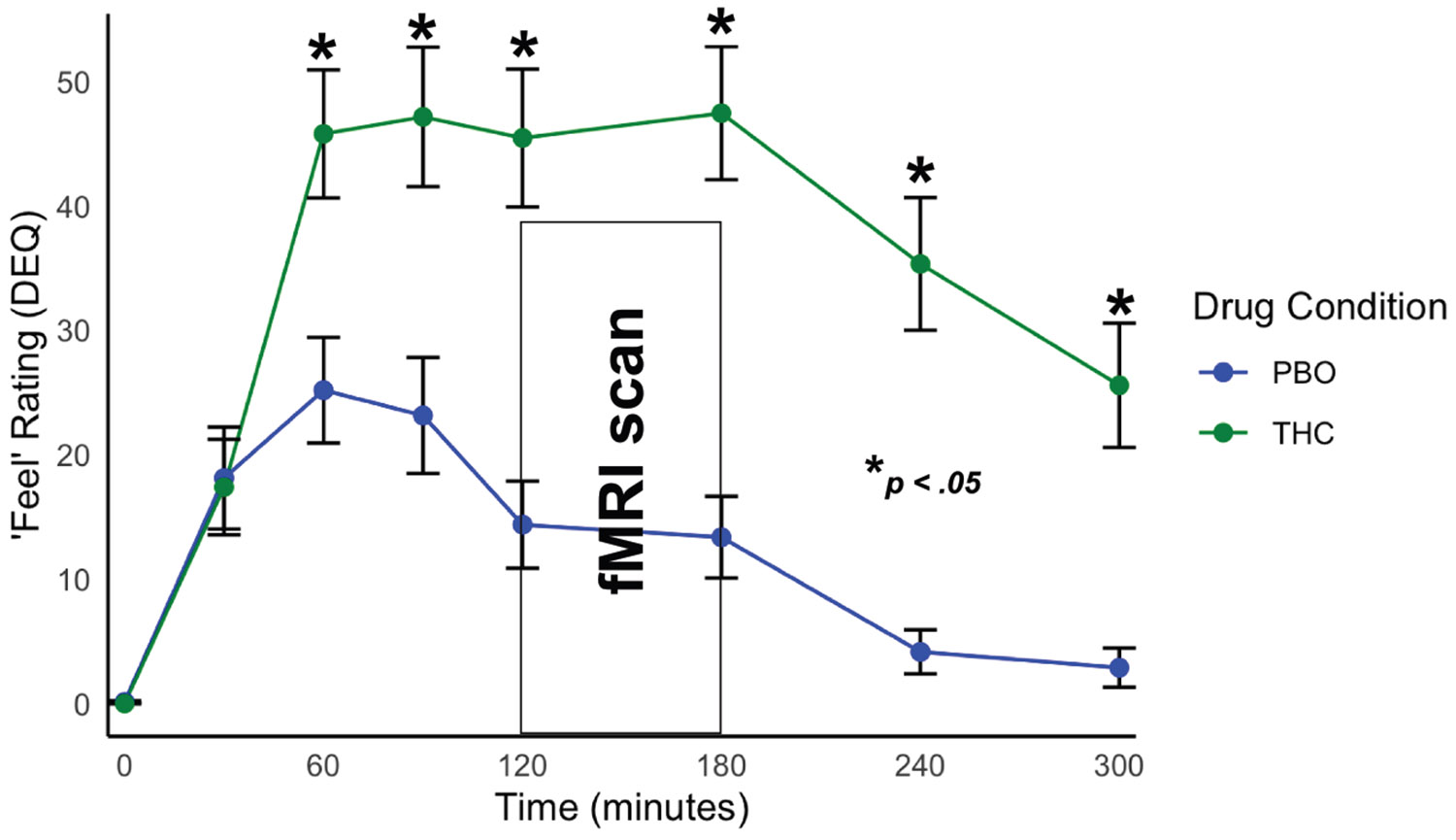
Subjective ratings of feel drug effect during Δ^9^-tetrahydrocannabinol (THC) and placebo (PBO) scan days. Participants endorsed elevated feel Drug Effects Questionnaire (DEQ) ratings during THC intoxication compared with PBO. Peak effects were experienced during the functional magnetic resonance imaging (fMRI) scan session.

**Figure 2.**
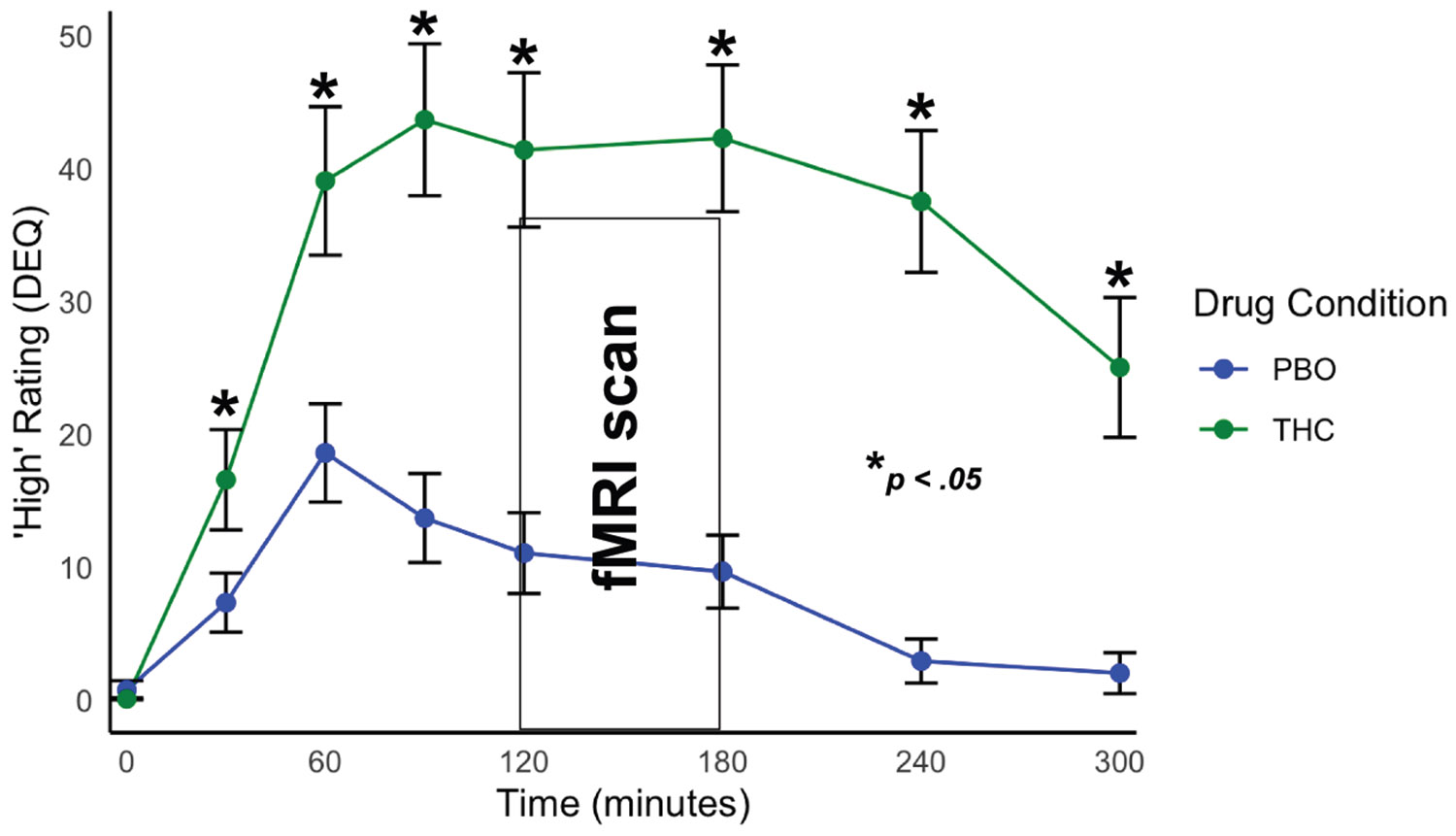
Subjective ratings of high during Δ^9^-tetrahydrocannabinol (THC) and placebo (PBO) scan days. Participants endorsed elevated high Drug Effects Questionnaire (DEQ) ratings during THC intoxication compared with PBO. Peak effects were experienced during the functional magnetic resonance imaging (fMRI) scan session.

THC significantly increased feel and high DEQ ratings compared with PBO (Figures 1 and 2). THC significantly increased participant reports of feeling any drug effects (THC: mean = 33.02, SD = 17.00; PBO: mean = 12.60, SD = 13.31; *t*_32_ = 6.71, *p* < .001) as well as how high they felt (THC: mean = 30.70, SD = 17.65; PBO: mean = 8.21, SD = 10.89; *t*_32_ = 6.40, *p* < .001).

### Intrinsic Connectivity Differences Across Frequency Bands When Comparing THC and PBO Sessions

**Figure 3.**
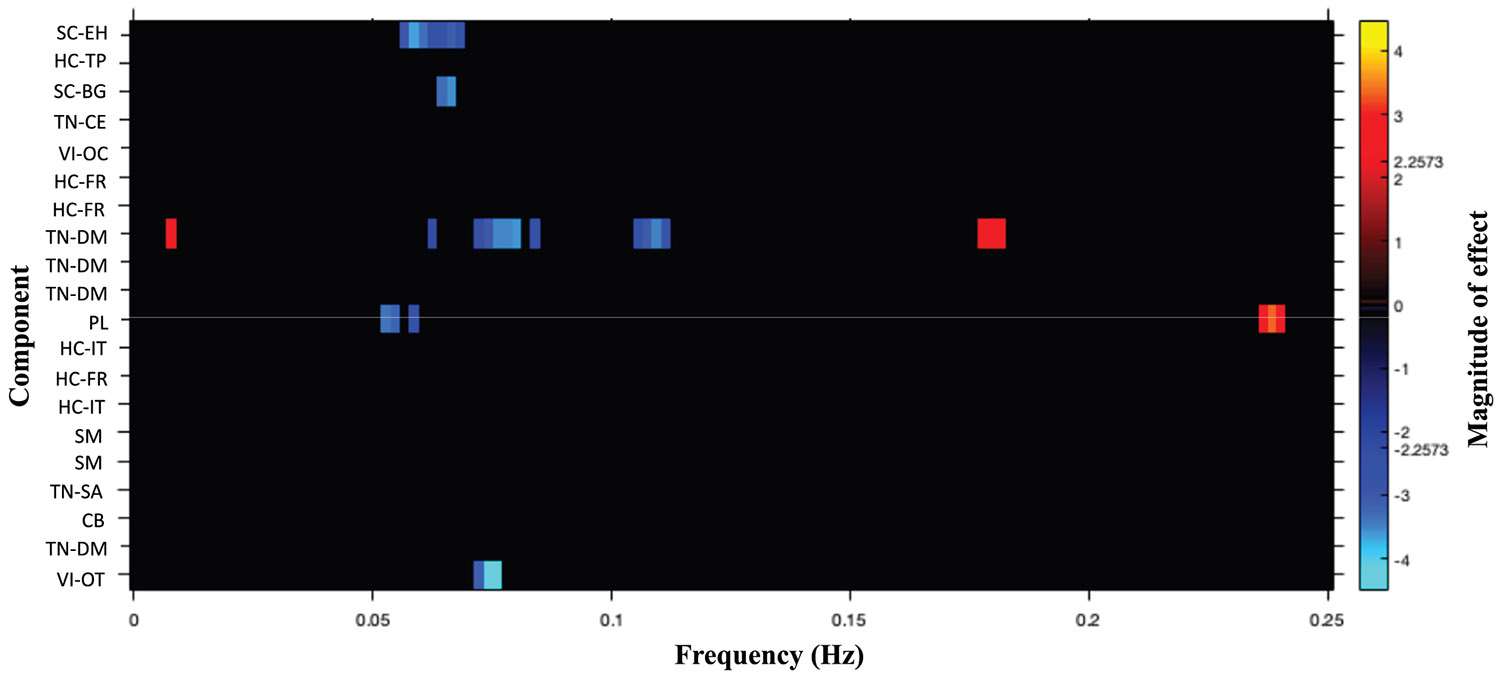
Δ^9^-Tetrahydrocannabinol (THC) increased and decreased connectivity across varying frequency bands (vs. placebo). Paired-samples *t* test results reveal differences in frequency bands across components for THC compared with placebo. Statistics reflect a combination of the magnitude and direction of each effect size calculated as –sign(*t*) × log_10_(*p* value). Cool colors suggest reductions, while hot colors suggest that increases in intrinsic connectivity were present. Presented effects passed a false discovery rate–corrected threshold (*p* < .05). BG, basal ganglia subdomain; CB, cerebellar domain; CE, central executive subdomain; DM, default mode subdomain; EH, extended hippocampal subdomain; FR, frontal subdomain; HC, higher cognition domain; IT, insular temporal subdomain; OC, occipital subdomain; OT, occipitotemporal subdomain; PL, paralimbic domain; SA, salience subdomain; SC, subcortical domain; SM, sensorimotor; TN, triple network domain; TP, temporoparietal subdomain; VI, visual domain.

**Figure 4.**
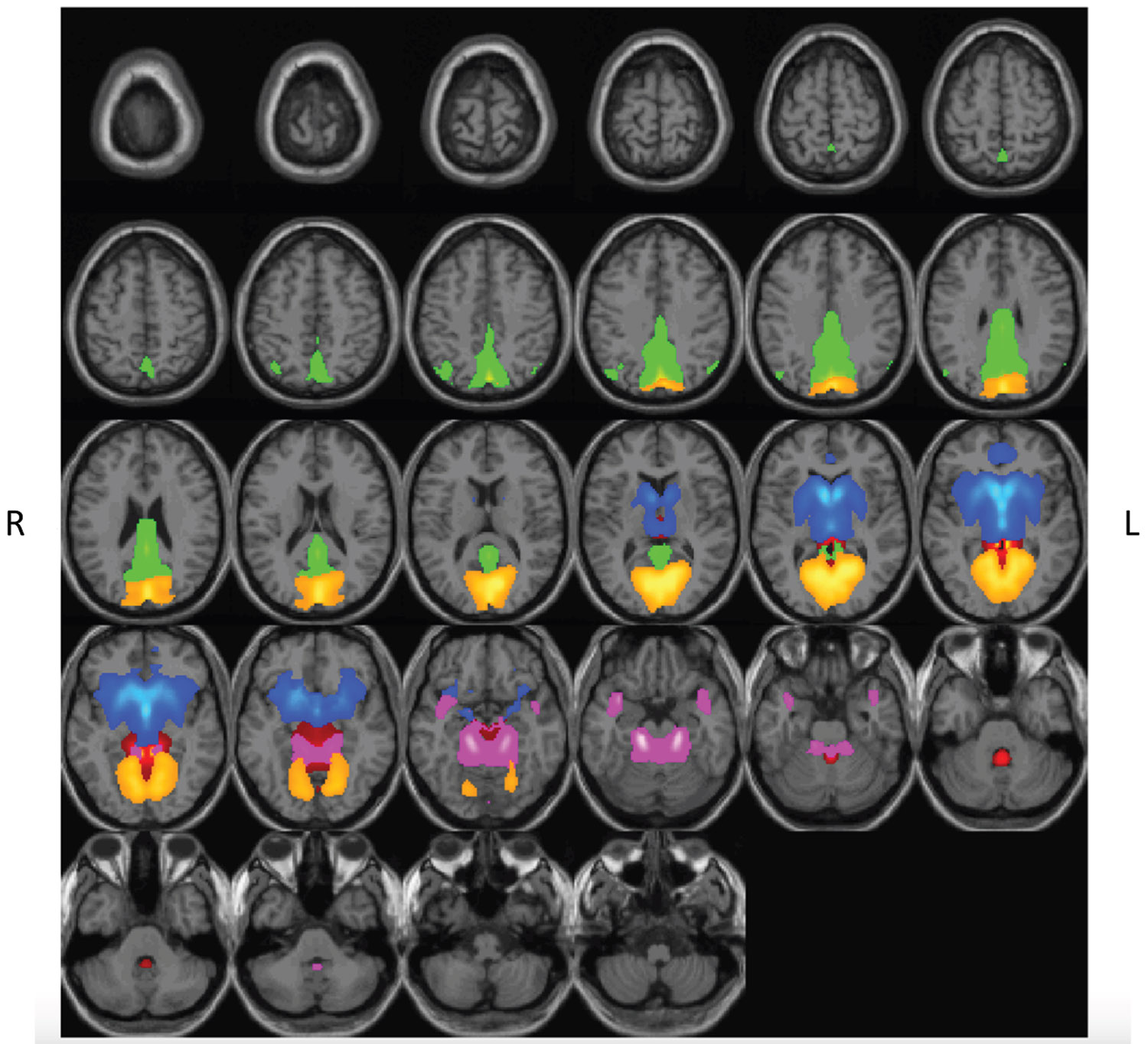
Spatial rendering of brain networks that display reductions in intrinsic connectivity during Δ^9^-tetrahydrocannabinol (THC) intoxication. Spatial rendering of functional networks where THC (vs. placebo) reduced intrinsic functional connectivity (0.05 < Hz < 0.12). Networks included subcortical domain–extended hippocampal subdomain (red), subcortical domain–basal ganglia subdomain (blue), triple network–default mode (green), paralimbic (pink), and visual domain–occipitotemporal subdomain (orange). Spatial components are thresholded (*t* > 2.0). L, left; R, right.

**Figure 5.**
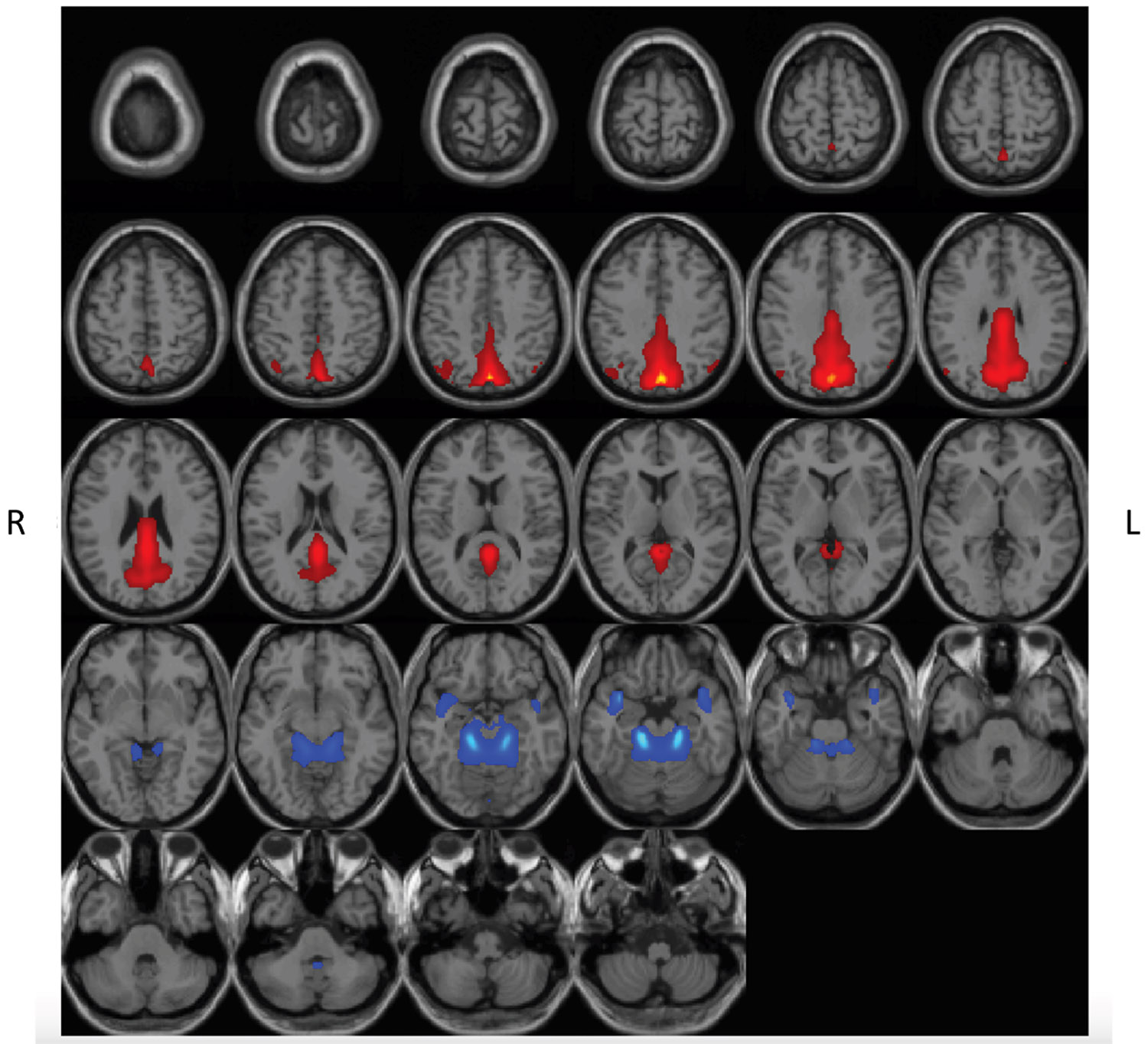
Spatial rendering of brain networks that display greater signal in high- and low-frequency bands during Δ^9^-tetrahydrocannabinol (THC) intoxication. Spatial rendering of functional networks where THC (vs. placebo) enhanced the presence of high- and low-frequency information (0.05 > Hz > 0.15). Networks included the triple network–default mode (red) and paralimbic (blue) domains. Spatial components are thresholded (*t* > 2.0). L, left; R, right.

We assessed for differences in rs-fMRI intrinsic connectivity based on an FDR-corrected (*p*_FDR_ < .05) paired *t* test comparing rs-fMRI scans after THC versus PBO. Because intrinsic connectivity within low frequency (<0.1 Hz) and high frequency (0.1 < Hz < 0.25) are linked to varied brain functions (61), we report differences across these frequency bands separately. The drug both increased and decreased spectral power, depending on the frequency band and component (see Figure 3). Specifically, spatial component 2 (subcortical domain–extended hippocampal subdomain [SC-EH]), component 5 (SC–basal ganglia subdomain [SC-BG]), component 13 (triple network–DM [TN-DM]), component 17 (PL), and component 30 (VI–occipitotemporal subdomain [VI-OT]) showed decreased rs-fMRI intrinsic connectivity within the frequency range 0.05 < Hz < 0.12 (spatial representations are shown in Figure 4). Spatial component 13 (TN-DM) and component 17 (PL) showed increased presence of BOLD frequencies in the following range: 0.05 > Hz > 0.12 (FDR-corrected threshold [*p*_FDR_ < .05]) (Figure 5).

### Between-Network Connectivity Differences Across Frequency Bands When Comparing Acute THC Intoxication and PBO Sessions

**Figure 6.**
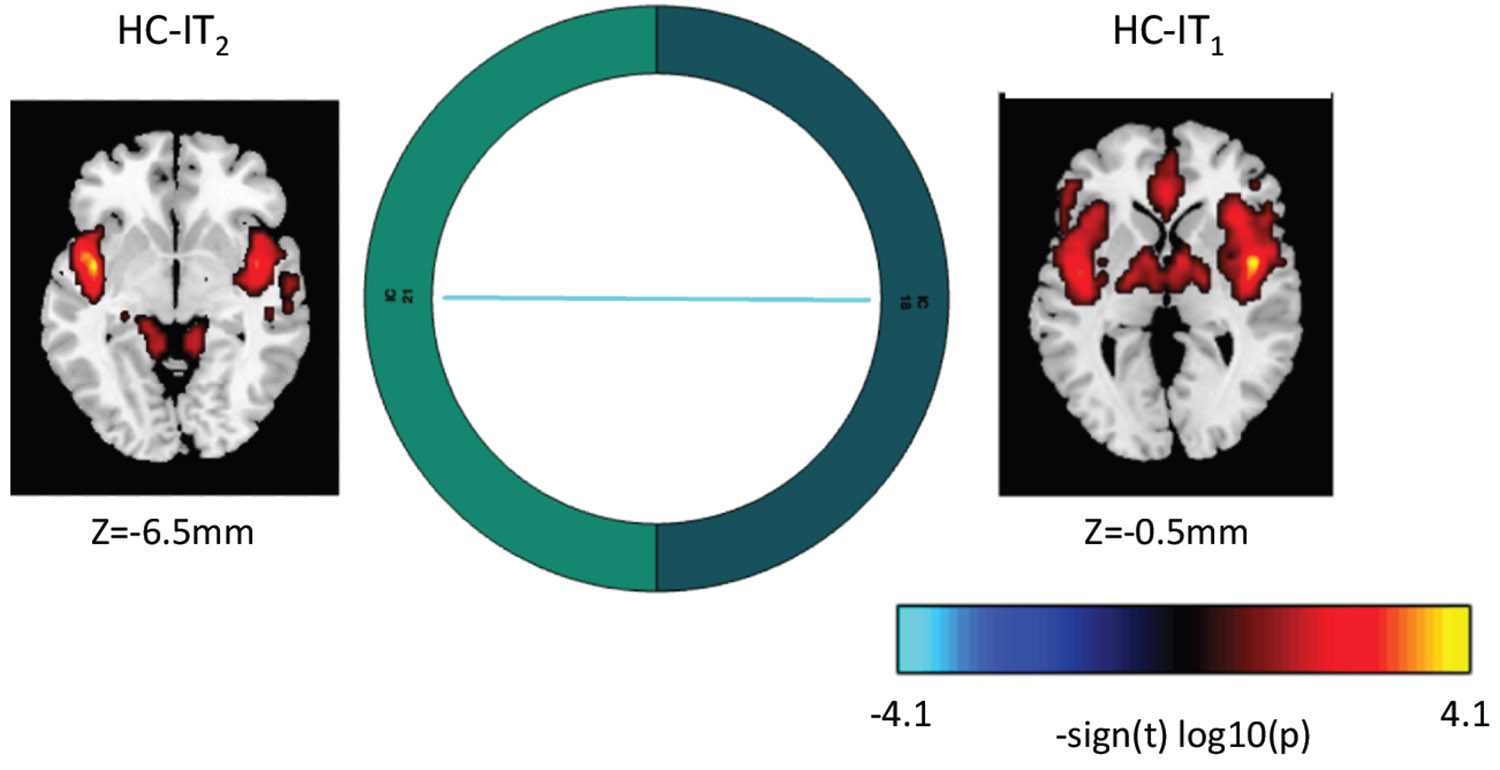
Δ^9^-Tetrahydrocannabinol (THC) reduced connectivity between an anterior cingulate–dorsal insula component (higher cognition domain–insular temporal subdomain [HC-IT_1_]) and a ventral insula–lingual gyrus component (HC-IT_2_) (vs. placebo). THC (vs. placebo) was associated with reduced connectivity between ventral and dorsal portions of the HC-IT network (*p* < .001, uncorrected).

We assessed for differences in functional network connectivity related to THC intoxication compared with PBO. We found reduced connectivity between component 18 (higher cognition domain–insular temporal subdomain [HC-IT_1_]) and component 21 (HC–insular temporal subdomain [HC-IT_2_]). This result (Figure 6) is based on an uncorrected threshold (*p* < .001).

### Past-Month Cannabis Use and Subjective Effects of THC Intoxication on rs-fMRI Intrinsic and Functional Network Connectivity

Past-month cannabis use frequency was not related to fMRI intrinsic or between-network connectivity in a multivariate analysis of covariance using an FDR-corrected (*p*_FDR_ < .05) or uncorrected (*p* < .001) threshold. Similarly, subjective effects of THC were not related to intrinsic or functional network connectivity using an FDR-corrected (*p*_FDR_ < .05) or uncorrected (*p* < .001) threshold. Finally, subjective feelings of euphoria, measured by the ARCI-MBG, were not related to intrinsic or functional network connectivity at an FDR-corrected (*p*_FDR_ < .05) or uncorrected (*p* < .001) threshold.

## DISCUSSION

In the current work, we found that THC (vs. PBO) reduced intrinsic connectivity in memory-, emotion-, and motivation-related circuitry (SC-EH, SC-BG). Networks associated with sensory processing, executive control, cognitive control, and self-reference, among others, were similarly affected (TN-DM, PL, VI-OT). Regions of interest within these networks are consistent with previous work (29) and support our hypothesis that THC impacts distributed brain networks. THC also increased high- and low-frequency signal (0.05 > Hz > 0.1) in TN-DM and PL networks. Functional connectivity findings in these frequency bands are interpreted in different ways, potentially characterizing clinically relevant disruptions in functional connectivity (40,61) or physiological noise and cellular processes indirectly related to mental experiences (63). With respect to functional network connectivity, we found that THC reduced connectivity between ventral and dorsal portions of the HC-IT network, which includes the anterior cingulate cortex, insula, and lingual gyrus. Decoupling of these overlapping networks could affect interoception and related processes. Finally, in contrast to previous work, subjective ratings of drug effects were not significantly related to functional differences in any of the extracted brain networks in the current sample. Taken together, our findings were largely consistent with previous literature (29).

Compared with PBO, THC reduced intrinsic connectivity in several networks. Reported reductions in frontostriatal (SC-EH, SC-BG) connectivity parallels existing literature (29), but unlike other reports (31,64), reduced frontostriatal connectivity was not related to subjective high or euphoria ratings. One reason for this could be the whole-brain network approach used in our study compared with specific seeds or individual circuits in previous work (29). More specific regional differences in activation or connectivity may be sensitive to subjective effects of THC. Another explanation may be our sample of occasional cannabis users, rather than nonusers or heavy users. Past work suggests that the effects of acute THC differs in occasional users (64) compared with noncannabis users and more regular cannabis users (31,64). Interestingly, however, in our study, cannabis use history was not significantly related to the effects of THC. It is possible that we were underpowered to assess this relationship, especially given the few nonusers or heavy users in the current sample.

THC reduced connectivity in other distributed brain networks that included regions in the occipital cortex, thalamus, parietal cortex, parahippocampus, temporal cortex, amygdala, and insula (TN-DM, PL, VI-OT). Disruptions in these networks could explain a variety of effects tied to THC intoxication (29,35). More specifically, abnormal sensory experiences are related to altered occipito-parahippocampal-thalamic, parahippocampal-temporal-insular, and visual network connectivity during THC intoxication (32,65). Such differences may also underlie cognitive impairment associated with THC, such as problems with episodic memory and spatial navigation (66). Reduced occipito-parahippocampal-thalamic and occipito-parietal network connectivity may also underlie maladaptive effects of THC on visuomotor function and executive control problems (67) as intrinsic connectivity across parietal, motor, and sensory regions are related to these processes (68). Furthermore, given the importance of thalamic activity in the integration of information from varied brain systems and sensory gating, disruptions in this network could account for the heightened intensity of perceptual experiences that have been previously associated with THC use (69).

THC reduced connectivity between a network composed of the anterior cingulate cortex and dorsal portions of the insula with a network composed of the ventral insula and lingual gyrus during THC intoxication. Functional brain changes in the insula have been linked to acute THC intoxication (70) and may correspond with the alterations in interoceptive experiences that individuals report when high (70). Furthermore, the involvement of the anterior cingulate and lingual gyrus may highlight pathways by which THC impacts executive function and related processes (66).

There are several limitations of the current work, which could be addressed by future studies. First, the current study has a relatively small, homogeneous sample, which reduces sources of variability related to mood, THC use history, or other factors that may alter the impact of THC on the brain. This lack of heterogeneity in the sample may be particularly important in explaining the lack of relationship between the effects of THC on subjective effects and ICA network changes. Future work might use larger and more heterogeneous samples to detect such effects. Next, the dose and type of THC used in the current study, while previously effective in work from our group (31), may be lower than that used in other studies and what cannabis users use on their own (71). Future work with a range of doses or just with a higher dose may reveal novel relationships between the brain and subjective feelings of THC intoxication. Furthermore, while dronabinol has some advantages, the current findings may not generalize to other cannabis products. Future work may explore additional varieties of cannabis. Third, there is question about the reliability of rs-fMRI data obtained from single scans (72,60). While ICA has been shown to extract reliable rs-fMRI networks, even from brief scans (73), the addition of task-related data or longer rs-fMRI scans could enhance future work. Another approach would be to monitor the time course of the effects of THC on brain function and subjective state. Fourth, the current sample did not include participants with current (and most lifetime) psychopathology. Few participants had any history of anxiety or depression. Future work should include such individuals to better understand how clinical symptoms interact with THC-related effects, particularly given the relationship between THC use and risk for psychopathology (8-14). Additional work may highlight biological pathways by which this vulnerability exists. Fifth, the study is limited by the methods used to quantify neural connectivity. Although GIG-ICA provides sensitive and reliable estimates of brain connectivity (73), multivariate prediction models may provide more nuanced relationships between the brain and subjective effects of THC (74).

### Conclusions

Our findings suggest that THC reduces network connectivity throughout the brain. It affects brain systems responsible for emotion, interoception, sensory processing, and executive function. Our findings expand on a growing body of work showing that THC affects distributed brain networks, including networks linked to many behavioral and perceptual changes that are reported with THC use (29,34,66). Future work is needed to build on our findings, which may better characterize risk factors for cannabis use disorder and related psychopathology. This could enhance prevention efforts and inform interventions tailored to THC-related problems.

## Supplementary Material

1

Supplementary material cited in this article is available online at https://doi.org/10.1016/j.bpsc.2025.09.005.

## Figures and Tables

**Table 1.** Demographic Characteristics of the Analytic Sample (*N* = 33)

Demographic Information	Values
Biological Sex	
Female	14
Male	19
Age, Years	22.2 ± 2.2
Ethnicity, Hispanic	9
Race	
Asian	5
Black	2
Multiracial	3
Native American/Alaska Native	1
Native Hawaiian/other Pacific Islander	0
Other	1
White	21
Current Substance Use	
Cannabis, days/month	6 ± 6.96 [0–25]
Cannabis, days/week	1.2 ± 1.6 [0–6]
Cannabis, days/lifetime	191.3 ± 224.5 [12–800]
Hazardous cannabis use, CUDIT-R score	4.94 ± 3.33 [0–15]
Alcohol, drinks/week	3.87 ± 3.50 [0–12.5]
Cigarettes	
Users, %	18%
Cigarettes/week among users	0.30 ± 1.13 [0.25–6.25]
CUD	
Current CUD	9%
Lifetime CUD	21%
Tested Positive for THC on Urine Drug Screen	30%
Reported ENDS Use in the Past Month	21%

Values are presented as *n*, mean ± SD, mean ± SD [range], or %.

CUD, cannabis use disorder; CUDIT-R, Cannabis Use Disorder Identification Test–Revised; ENDS, electronic nicotine delivery system; THC, Δ^9^-tetrahydrocannabinol.

## References

[1] Patrick ME, Miech RA, Pang YC, Leventhal AM, Harlow AF (2025): Use of Delta-8-THC and other types of cannabis among young adults in the U.S. Am J Prev Med 68:1179–1181. 40024583 10.1016/j.amepre.2025.02.009 PMC12092172

[2] Volkow ND, Wise RA, Baler R (2017): The dopamine motive system: Implications for drug and food addiction. Nat Rev Neurosci 18:741–752. 29142296 10.1038/nrn.2017.130

[3] Crean RD, Crane NA, Mason BJ (2011): An evidence based review of acute and long-term effects of cannabis use on executive cognitive functions. J Addict Med 5:1–8. 21321675 10.1097/ADM.0b013e31820c23fa PMC3037578

[4] Sevigny EL (2013): Is today’s marijuana more potent simply because it’s fresher? Drug Test Anal 5:62–67. 23169764 10.1002/dta.1430

[5] Chandra S, Radwan MM, Majumdar CG, Church JC, Freeman TP, ElSohly MA (2019): New trends in cannabis potency in USA and Europe during the last decade (2008–2017). Eur Arch Psychiatry Clin Neurosci 269:5–15. 30671616 10.1007/s00406-019-00983-5

[6] ElSohly MA, Chandra S, Radwan M, Majumdar CG, Church JC (2021): A comprehensive review of cannabis potency in the United States in the last decade. Biol Psychiatry Cogn Neurosci Neuroimaging 6:603–606. 33508497 10.1016/j.bpsc.2020.12.016

[7] Caprari C, Ferri E, Schmid MG, Del Mercato LL, Citti C, Cannazza G (2024): Δ9-Tetrahydrocannabiphorol: Identification and quantification in recreational products. Forensic Chem 40:100595.

[8] Patton GC, Coffey C, Carlin JB, Degenhardt L, Lynskey M, Hall W (2002): Cannabis use and mental health in young people: Cohort study. BMJ 325:1195–1198. 12446533 10.1136/bmj.325.7374.1195 PMC135489

[9] Casadio P, Fernandes C, Murray RM, Di Forti M (2011): Cannabis use in young people: The risk for schizophrenia. Neurosci Biobehav Rev 35:1779–1787. 21530584 10.1016/j.neubiorev.2011.04.007

[10] Bechtold J, Hipwell A, Lewis DA, Loeber R, Pardini D (2016): Concurrent and sustained cumulative effects of adolescent marijuana use on subclinical psychotic symptoms. Am J Psychiatry 173:781–789. 27138587 10.1176/appi.ajp.2016.15070878 PMC5390686

[11] Hasin DS, Kerridge BT, Saha TD, Huang B, Pickering R, Smith SM, (2016): Prevalence and correlates of DSM-5 cannabis use disorder, 2012–2013: Findings from the national epidemiologic survey on alcohol and related conditions–III. Am J Psychiatry 173:588–599. 26940807 10.1176/appi.ajp.2015.15070907 PMC5026387

[12] De Aquino JP, Sherif M, Radhakrishnan R, Cahill JD, Ranganathan M, D’Souza DC (2018): The psychiatric consequences of cannabinoids. Clin Ther 40:1448–1456. 29678279 10.1016/j.clinthera.2018.03.013

[13] Han BH, Palamar JJ (2020): Trends in cannabis use among older adults in the United States, 2015–2018. JAMA Intern Med 180:609–611. 32091531 10.1001/jamainternmed.2019.7517 PMC7042817

[14] Scheim AI, Maghsoudi N, Marshall Z, Churchill S, Ziegler C, Werb D (2020): Impact evaluations of drug decriminalisation and legal regulation on drug use, health and social harms: A systematic review. BMJ Open 10:e035148. 10.1136/bmjopen-2019-035148 PMC7507857 32958480

[15] Bloomfield MAP, Ashok AH, Volkow ND, Howes OD (2016): The effects of Δ9-tetrahydrocannabinol on the dopamine system. Nature 539:369–377. 27853201 10.1038/nature20153 PMC5123717

[16] Bossong MG, Mehta MA, van Berckel BNM, Howes OD, Kahn RS, Stokes PRA (2015): Further human evidence for striatal dopamine release induced by administration of Δ9-tetrahydrocannabinol (THC): Selectivity to limbic striatum. Psychopharmacol (Berl) 232:2723–2729. 10.1007/s00213-015-3915-0 PMC4816196 25801289

[17] Bossong MG, van Berckel BNM, Boellaard R, Zuurman L, Schuit RC, Windhorst AD, (2009): Δ9-tetrahydrocannabinol induces dopamine release in the human striatum. Neuropsychopharmacology 34:759–766. 18754005 10.1038/npp.2008.138

[18] Fernández-Ruiz J, Moreno-Martet M, Rodríguez-Cueto C, Palomo-Garo C, Gómez-Cañas M, Valdeolivas S, (2011): Prospects for cannabinoid therapies in basal ganglia disorders. Br J Pharmacol 163:1365–1378. 21545415 10.1111/j.1476-5381.2011.01365.x PMC3165947

[19] Chen JP, Paredes W, Li J, Smith D, Lowinson J, Gardner EL (1990): Delta 9-tetrahydrocannabinol produces naloxone-blockable enhancement of presynaptic basal dopamine efflux in nucleus accumbens of conscious, freely-moving rats as measured by intracerebral microdialysis. Psychopharmacol (Berl) 102:156–162. 10.1007/BF02245916 2177204

[20] French ED, Dillon K, Wu X (1997): Cannabinoids excite dopamine neurons in the ventral tegmentum and substantia nigra. NeuroReport 8:649–652. 9106740 10.1097/00001756-199702100-00014

[21] Tanda G, Pontieri FE, Di Chiara G (1997): Cannabinoid and heroin activation of mesolimbic dopamine transmission by a common mu1 opioid receptor mechanism. Science 276:2048–2050. 9197269 10.1126/science.276.5321.2048

[22] Gessa GL, Melis M, Muntoni AL, Diana M (1998): Cannabinoids activate mesolimbic dopamine neurons by an action on cannabinoid CB1 receptors. Eur J Pharmacol 341:39–44. 9489854 10.1016/s0014-2999(97)01442-8

[23] Ramaekers JG, Mason NL, Toennes SW, Theunissen EL, Amico E (2022): Functional brain connectomes reflect acute and chronic cannabis use. Sci Rep 12:2449. 35165360 10.1038/s41598-022-06509-9 PMC8844352

[24] Weinstein A, Livny A, Weizman A (2016): Brain imaging studies on the cognitive, pharmacological and neurobiological effects of cannabis in humans: Evidence from studies of adult users. Curr Pharm Des 22:6366–6379. 27549374 10.2174/1381612822666160822151323

[25] Crane NA, Wade NE (2023): Cannabis and neuropsychology. In: Brown GG, King TZ, Haaland KY, Crosson B, editors. APA Handbook of Neuropsychology, Volume 1: Neurobehavioral Disorders and Conditions: Accepted Science and Open Questions. Washington: American Psychological Association, 627–647.

[26] Pessoa L (2018): Understanding emotion with brain networks. Curr Opin Behav Sci 19:19–25. 29915794 10.1016/j.cobeha.2017.09.005 PMC6003711

[27] Fox MD, Greicius M (2010): Clinical applications of resting state functional connectivity. Front Syst Neurosci 4:19. 20592951 10.3389/fnsys.2010.00019 PMC2893721

[28] Thomson H, Labuschagne I, Greenwood L-M, Robinson E, Sehl H, Suo C, Lorenzetti V (2022): Is resting-state functional connectivity altered in regular cannabis users? A systematic review of the literature. Psychopharmacol (Berl) 239:1191–1209. 10.1007/s00213-021-05938-0 34415377

[29] Lorenzetti V, Gaillard A, Thomson D, Englund A, Freeman TP (2023): Effects of cannabinoids on resting state functional brain connectivity: A systematic review. Neurosci Biobehav Rev 145:105014. 36563921 10.1016/j.neubiorev.2022.105014

[30] Mason NL, Theunissen EL, Hutten NRPW, Tse DHY, Toennes SW, Stiers P, Ramaekers JG (2019): Cannabis induced increase in striatal glutamate associated with loss of functional corticostriatal connectivity. Eur Neuropsychopharmacol 29:247–256. 30553697 10.1016/j.euroneuro.2018.12.003

[31] Crane NA, Phan KL (2021): Effect of Δ9-tetrahydrocannabinol on frontostriatal resting state functional connectivity and subjective euphoric response in healthy young adults. Drug Alcohol Depend 221:108565. 33592558 10.1016/j.drugalcdep.2021.108565 PMC8026570

[32] Klumpers F, Denys D, Kenemans JL, Grillon C, van der Aart J, Baas JMP (2012): Testing the effects of Δ9-THC and D-cycloserine on extinction of conditioned fear in humans. J Psychopharmacol 26:471–478. 22351380 10.1177/0269881111431624 PMC3454470

[33] Rzepa E, Tudge L, McCabe C (2015): The CB1 neutral antagonist tetrahydrocannabivarin reduces default mode network and increases executive control network resting state functional connectivity in healthy volunteers. Int J Neuropsychopharmacol 19:pyv092. 26362774 10.1093/ijnp/pyv092 PMC4772823

[34] Bossong MG, van Hell HH, Schubart CD, van Saane W, Iseger TA, Jager G, (2019): Acute effects of Δ9-tetrahydrocannabinol (THC) on resting state brain function and their modulation by COMT genotype. Eur Neuropsychopharmacol 29:766–776. 30975584 10.1016/j.euroneuro.2019.03.010

[35] Wall MB, Pope R, Freeman TP, Kowalczyk OS, Demetriou L, Mokrysz C, (2019): Dissociable effects of cannabis with and without cannabidiol on the human brain’s resting-state functional connectivity. J Psychopharmacol 33:822–830. 31013455 10.1177/0269881119841568

[36] Zaytseva Y, Horáček J, Hlinka J, Fajnerová I, Androvičová R, Tintěra J, (2019): Cannabis-induced altered states of consciousness are associated with specific dynamic brain connectivity states. J Psychopharmacol 33:811–821. 31154891 10.1177/0269881119849814

[37] Du Y, Allen EA, He H, Sui J, Wu L, Calhoun VD (2016): Artifact removal in the context of group ICA: A comparison of single-subject and group approaches. Hum Brain Mapp 37:1005–1025. 26859308 10.1002/hbm.23086 PMC5784424

[38] Du Y, Fan Y (2013): Group information guided ICA for fMRI data analysis. Neuroimage 69:157–197. 23194820 10.1016/j.neuroimage.2012.11.008

[39] Salman MS, Du Y, Lin D, Fu Z, Fedorov A, Damaraju E, (2019): Group ICA for identifying biomarkers in schizophrenia: ‘Adaptive’ networks via spatially constrained ICA show more sensitivity to group differences than spatio-temporal regression. Neuroimage Clin 22:101747. 30921608 10.1016/j.nicl.2019.101747 PMC6438914

[40] Calhoun VD, Sui J, Kiehl K, Turner JA, Allen EA, Pearlson G (2011): Exploring the psychosis functional connectome: Aberrant intrinsic networks in schizophrenia and bipolar disorder. Front Psychiatry 2:75. 22291663 10.3389/fpsyt.2011.00075 PMC3254121

[41] Calhoun VD, Liu J, Adalı T (2009): A review of group ICA for fMRI data and ICA for joint inference of imaging, genetic, and ERP data. Neuroimage 45(suppl):S163–S172. 19059344 10.1016/j.neuroimage.2008.10.057 PMC2651152

[42] Johanson CE, Uhlenhuth EH (1980): Drug preference and mood in humans: d-amphetamine. Psychopharmacology 71:275–279. 6779335 10.1007/BF00433062

[43] Martin WR, Sloan JW, Sapira JD, Jasinski DR (1971): Physiologic, subjective, and behavioral effects of amphetamine, methamphetamine, ephedrine, phenmetrazine, and methylphenidate in man. Clin Pharmacol Ther 12:245–258. 5554941 10.1002/cpt1971122part1245

[44] Justice AJH, de Wit H (1999): Acute effects of d-amphetamine during the follicular and luteal phases of the menstrual cycle in women. Psychopharmacol (Berl) 145:67–75. 10.1007/s002130051033 10445374

[45] Mayo LM, De Wit H (2015): Acquisition of responses to a methamphetamine-associated cue in healthy humans: Self-report, behavioral, and psychophysiological measures. Neuropsychopharmacology 40:1734–1741. 25601231 10.1038/npp.2015.21 PMC4915257

[46] Esteban O, Markiewicz CJ, Blair RW, Moodie CA, Isik AI, Erramuzpe A, (2019): fMRIPrep: A robust preprocessing pipeline for functional MRI. Nat Methods 16:111–116. 30532080 10.1038/s41592-018-0235-4 PMC6319393

[47] Esteban O, Blair RW, Markiewicz CJ, Berleant SL, Moodie CA, Ma F, (2018): FMRIPrep. Zenodo. Available at: 10.5281/zenodo.852659. Accessed October 8, 2024.

[48] Gorgolewski K, Burns CD, Madison C, Clark D, Halchenko YO, Waskom ML, Ghosh SS (2011): Nipype: A flexible, lightweight and extensible neuroimaging data processing framework in python. Front Neuroinform 5:13. 21897815 10.3389/fninf.2011.00013 PMC3159964

[49] Gorgolewski KJ, Esteban O, Markiewicz CJ, Ziegler E, Ellis DG, Notter MP, (2018): Nipype. Available at: 10.5281/zenodo.596855. Accessed October 8, 2024.

[50] Calhoun V (2004): GIFT. Available at: https://trendscenter.org/software/gift/. Accessed September 16, 2024.

[51] Griffanti L, Douaud G, Bijsterbosch J, Evangelisti S, Alfaro-Almagro F, Glasser MF, (2017): Hand classification of fMRI ICA noise components. Neuroimage 154:188–205. 27989777 10.1016/j.neuroimage.2016.12.036 PMC5489418

[52] Allen EA, Erhardt EB, Damaraju E, Gruner W, Segall JM, Silva RF, (2011): A baseline for the multivariate comparison of resting-state networks. Front Syst Neurosci 5:2. 21442040 10.3389/fnsys.2011.00002 PMC3051178

[53] Jensen KM, Turner JA, Uddin LQ, Calhoun VD, Iraji A (2024): Addressing inconsistency in functional neuroimaging: A replicable data-driven multi-scale functional atlas for canonical brain networks. bioRxiv 10.1101/2024.09.09.612129.

[54] Du Y, Fu Z, Sui J, Gao S, Xing Y, Lin D, (2020): NeuroMark: An automated and adaptive ICA based pipeline to identify reproducible fMRI markers of brain disorders. Neuroimage Clin 28:102375. 32961402 10.1016/j.nicl.2020.102375 PMC7509081

[55] Braga RM, Van Dijk KRAV, Polimeni JR, Eldaief MC, Buckner RL (2019): Parallel distributed networks resolved at high resolution reveal close juxtaposition of distinct regions. J Neurophysiol 121:1513–1534. 30785825 10.1152/jn.00808.2018 PMC6485740

[56] Yeo BTT, Krienen FM, Sepulcre J, Sabuncu MR, Lashkari D, Hollinshead M, (2011): The organization of the human cerebral cortex estimated by intrinsic functional connectivity. J Neurophysiol 106:1125–1165. 21653723 10.1152/jn.00338.2011 PMC3174820

[57] Li K, Fu Z, Luo X, Zeng Q, Huang P, Zhang M, Vince CD (2021): The influence of cerebral small vessel disease on static and dynamic functional network connectivity in subjects along Alzheimer’s disease continuum. Brain Connect 11:189–200. 33198482 10.1089/brain.2020.0819 PMC8080908

[58] Fu Z, Sui J, Turner JA, Du Y, Assaf M, Pearlson GD, Calhoun VD (2021): Dynamic functional network reconfiguration underlying the pathophysiology of schizophrenia and autism spectrum disorder. Hum Brain Mapp 42:80–94. 32965740 10.1002/hbm.25205 PMC7721229

[59] Fu Z, Iraji A, Turner JA, Sui J, Miller R, Pearlson GD, Calhoun VD (2021): Dynamic state with covarying brain activity-connectivity: On the pathophysiology of schizophrenia. Neuroimage 224:117385. 32950691 10.1016/j.neuroimage.2020.117385 PMC7781150

[60] Elliott ML, Knodt AR, Ireland D, Morris ML, Poulton R, Ramrakha S, (2020): What is the test–retest reliability of common task-functional MRI measures? New empirical evidence and a meta-analysis. Psychol Sci 31:792–806. 32489141 10.1177/0956797620916786 PMC7370246

[61] Tadayonnejad R, Yang S, Kumar A, Ajilore O (2015): Clinical, cognitive, and functional connectivity correlations of resting-state intrinsic brain activity alterations in unmedicated depression. J Affect Disord 172:241–250. 25451423 10.1016/j.jad.2014.10.017 PMC4402240

[62] Bullmore E, Sporns O (2009): Complex brain networks: Graph theoretical analysis of structural and functional systems. Nat Rev Neurosci 10:186–198. 19190637 10.1038/nrn2575

[63] Zuo X-N, Di Martino A, Kelly C, Shehzad ZE, Gee DG, Klein DF, (2010): The oscillating brain: Complex and reliable. Neuroimage 49:1432–1445. 19782143 10.1016/j.neuroimage.2009.09.037 PMC2856476

[64] Mason NL, Theunissen EL, Hutten NRPW, Tse DHY, Toennes SW, Jansen JFA, (2021): Reduced responsiveness of the reward system is associated with tolerance to cannabis impairment in chronic users. Addict Biol 26:e12870. 31865628 10.1111/adb.12870 PMC7757162

[65] Winton-Brown TT, Allen P, Bhattacharyya S, Borgwardt SJ, Fusar-Poli P, Crippa JA, (2011): Modulation of auditory and visual processing by delta-9-tetrahydrocannabinol and cannabidiol: An fMRI study. Neuropsychopharmacology 36:1340–1348. 21412224 10.1038/npp.2011.17 PMC3096803

[66] Burggren AC, Shirazi A, Ginder N, London ED (2019): Cannabis effects on brain structure, function, and cognition: Considerations for medical uses of cannabis and its derivatives. Am J Drug Alcohol Abuse 45:563–579. 31365275 10.1080/00952990.2019.1634086 PMC7027431

[67] Shrivastava A, Johnston M, Tsuang M (2011): Cannabis use and cognitive dysfunction. Indian J Psychiatry 53:187–191. 22135433 10.4103/0019-5545.86796 PMC3221171

[68] Seitzman BA, Snyder AZ, Leuthardt EC, Shimony JS (2019): The state of resting state networks. Top Magn Reson Imaging 28:189–196. 31385898 10.1097/RMR.0000000000000214 PMC6686880

[69] Vukadinovic Z, Herman MS, Rosenzweig I (2013): Cannabis, psychosis and the thalamus: A theoretical review. Neurosci Biobehav Rev 37:658–667. 23458778 10.1016/j.neubiorev.2013.02.013

[70] van Hell HH, Bossong MG, Jager G, Kristo G, van Osch MJP, Zelaya F, (2011): Evidence for involvement of the insula in the psychotropic effects of THC in humans: A double-blind, randomized pharmacological MRI study. Int J Neuropsychopharmacol 14:1377–1388. 21489346 10.1017/S1461145711000526

[71] Cupo L, Plitman E, Guma E, Chakravarty MM (2021): A systematic review of neuroimaging and acute cannabis exposure in age-of-risk for psychosis. Transl Psychiatry 11:217. 33850098 10.1038/s41398-021-01295-w PMC8044224

[72] Marek S, Tervo-Clemmens B, Calabro FJ, Montez DF, Kay BP, Hatoum AS, (2022): Reproducible brain-wide association studies require thousands of individuals. Nature 603:654–660. 35296861 10.1038/s41586-022-04492-9 PMC8991999

[73] Duda M, Iraji A, Ford JM, Lim KO, Mathalon DH, Mueller BA, (2023): Reliability and clinical utility of spatially constrained estimates of intrinsic functional networks from very short fMRI scans. Hum Brain Mapp 44:2620–2635. 36840728 10.1002/hbm.26234 PMC10028646

[74] Kragel PA, Han X, Kraynak TE, Gianaros PJ, Wager TD (2021): Functional MRI can be highly reliable, but it depends on what you measure: A commentary on Elliott et al. (2020). Psychol Sci 32:622–626. 33685310 10.1177/0956797621989730 PMC8258303

