# Supplementary material for: Δ9-Tetrahydrocannabinol Alters Limbic and Frontal Functional Brain Connectomes Among Young Adult Cannabis Users
Source: Biol Psychiatry Cogn Neurosci Neuroimaging. Author manuscript; Available in PMC 2026 May 17. (PMC13180306; doi:10.1016/j.bpsc.2025.09.005)
Supplement: 1 [file NIHMS2173398-supplement-1.pdf]

SUPPLEMENTARY INFORMATION

Δ^9-Tetrahydrocannabinol Alters Limbic and Frontal Functional Brain Connectomes Among Young Adult Cannabis Users

Anderson *et al.*

S1. fMRI prep related preprocessing methods.

1.1.Anatomical data preprocessing

Each T1w image was corrected for intensity non-uniformity (INU) with N4BiasFieldCorrection (1), distributed with ANTs 2.5.0 (2). The T1w-reference was then skull-stripped with a Nipype implementation of the antsBrainExtraction.sh workflow (from ANTs), using OASIS30ANTs as target template. An anatomical T1w-reference map was computed after registration of the T1w images (after INU-correction) using mri_robust_template (3). Volume-based spatial normalization to one standard space (MNI152NLin2009cAsym) was performed through nonlinear registration with antsRegistration (ANTs 2.5.0), using brain-extracted versions of both T1w reference and the T1w template. The following template was selected for spatial normalization and accessed with TemplateFlow (4); ICBM 152 nonlinear asymmetrical template version 2009c (5).

1.2.Functional data preprocessing

First, a reference volume was generated, using a custom methodology of fMRIPrep, for use in head motion correction. Head-motion parameters with respect to the BOLD reference (transformation matrices, and six corresponding rotation and translation parameters) are estimated before any spatiotemporal filtering using mcflirt (6). Participants did not differ in average head motion in THC relative to placebo sessions (t=-1.79, p=0.083). The BOLD reference was then co-registered to the T1w reference using mri_coreg (FreeSurfer) followed by FLIRT (7) with the boundary-based registration (8) cost-function. Co-registration was configured with six degrees of freedom. Several confounding time-series were calculated based on the preprocessed BOLD: framewise displacement (FD) and three region-wise global signals. FD is calculated for each functional run using implementations in Nipype (9). Many internal

operations of fMRIPrep use Nilearn 0.10.2 (10), mostly within the functional processing workflow. For more details of the pipeline, see the section corresponding to workflows in fMRIPrep's documentation. Preprocessed data were smoothed using a 5mm FWHM kernel.

S2. Spatial maps and corresponding frequency spectra for all 30 original group-information guided independent component analysis components.

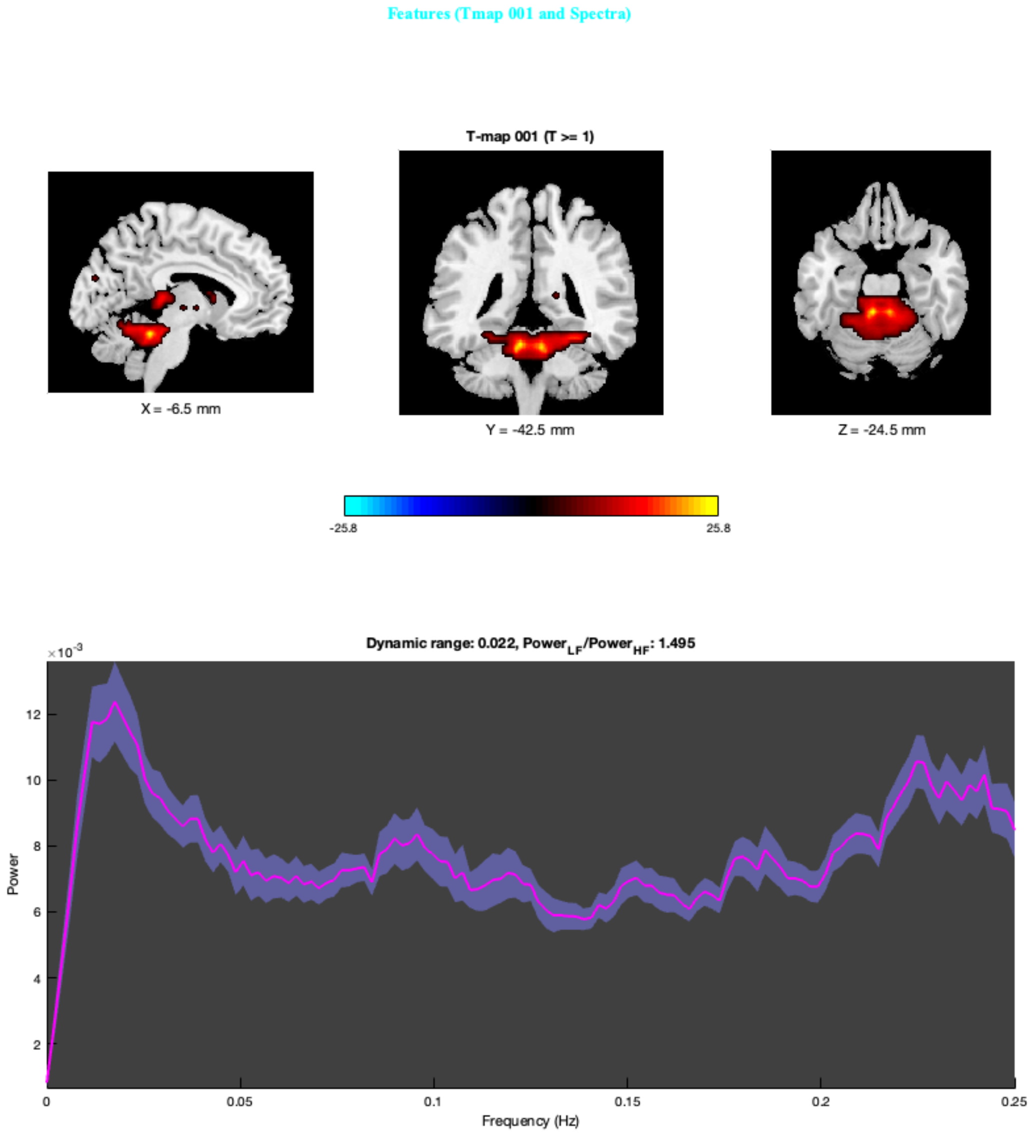

Features (Tmap 002 and Spectra)

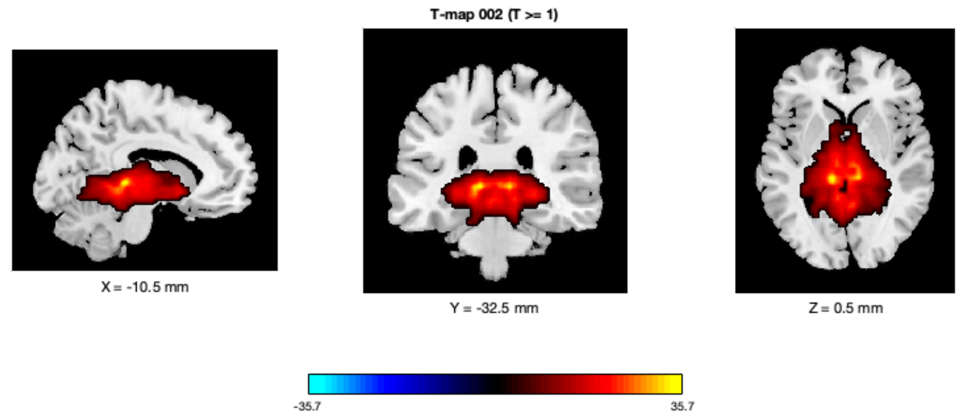

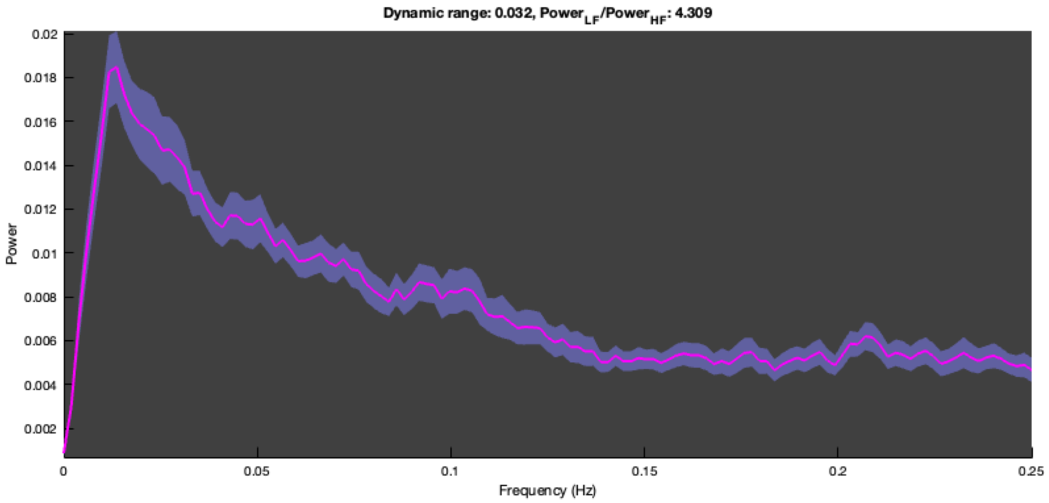

Features (Tmap 003 and Spectra)

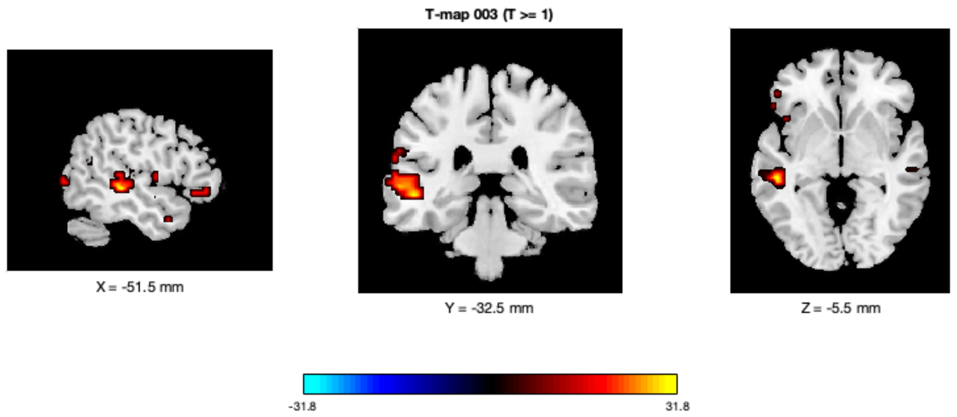

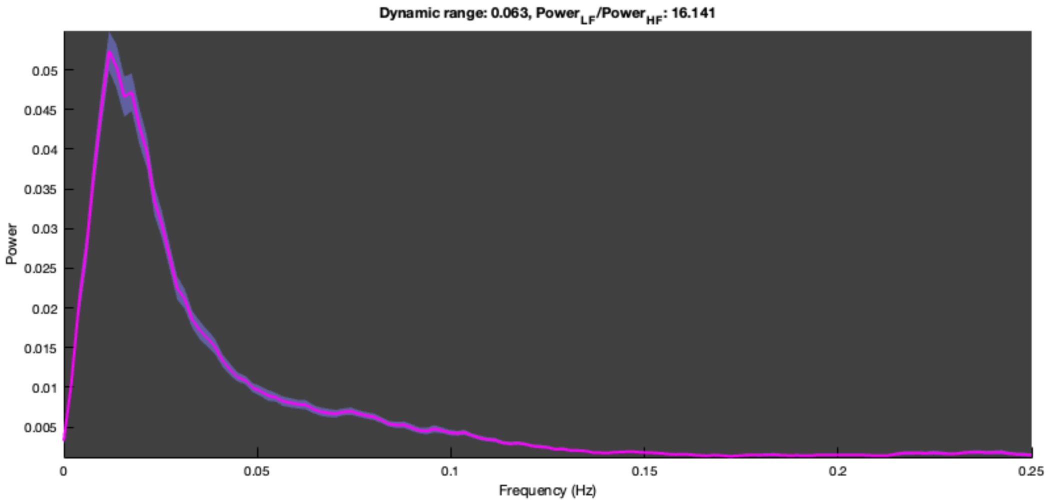

Features (Tmap 004 and Spectra)

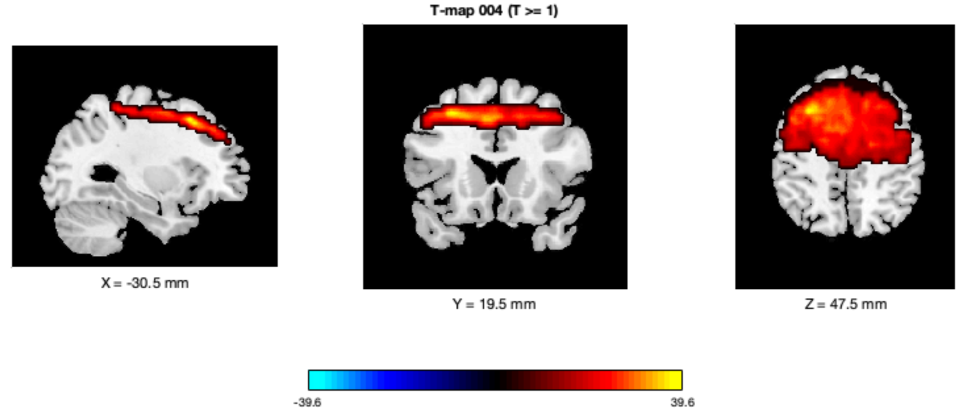

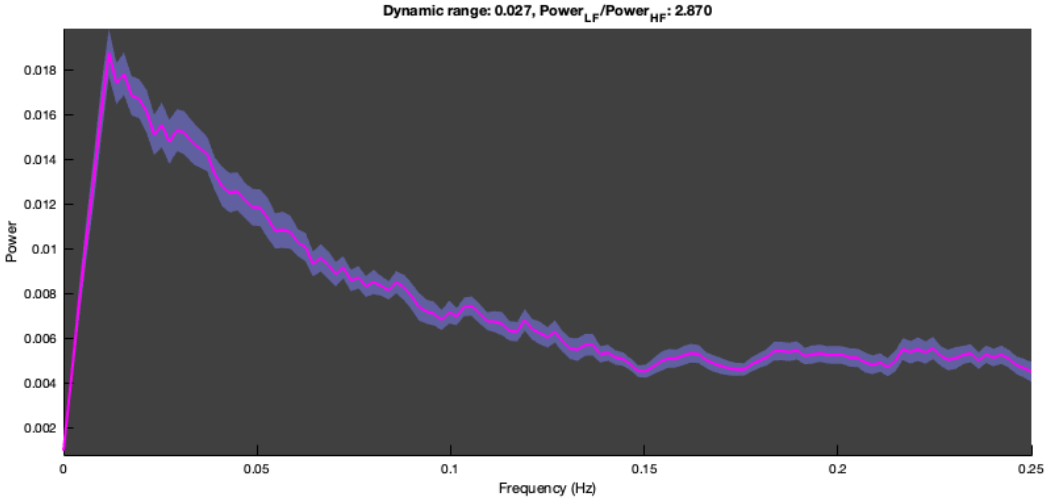

Features (Tmap 005 and Spectra)

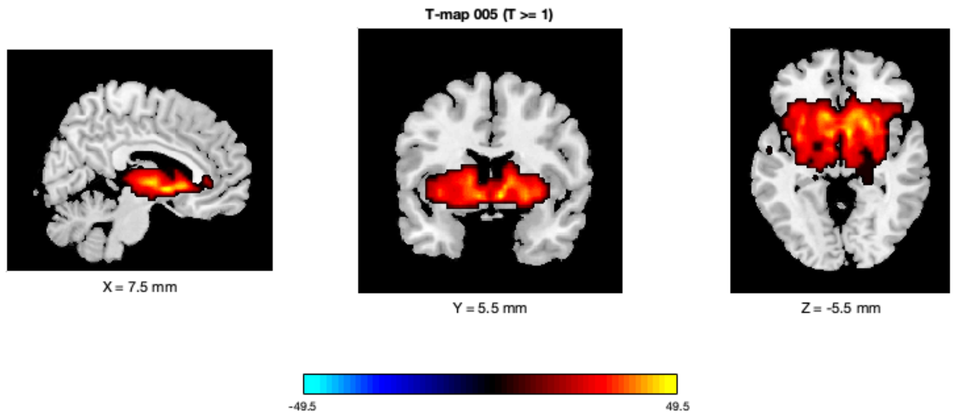

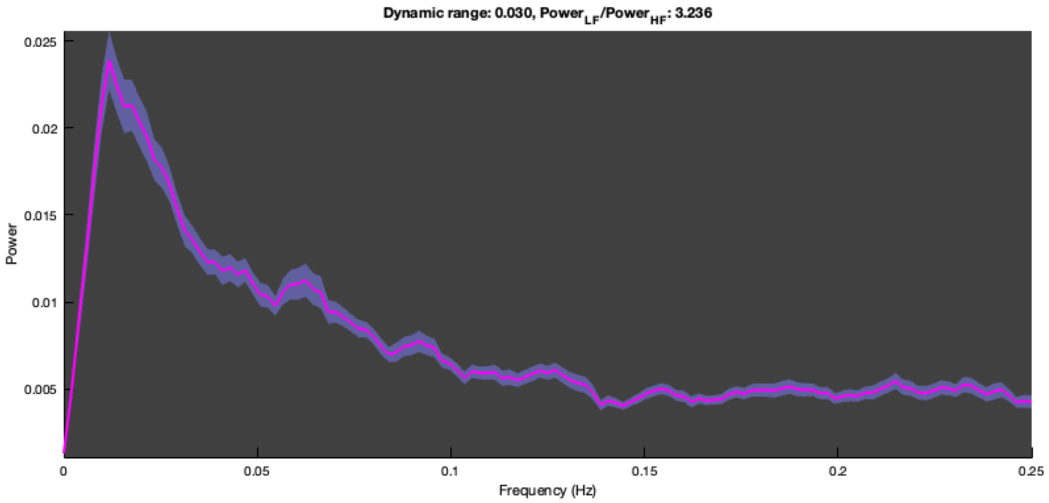

Features (Tmap 006 and Spectra)

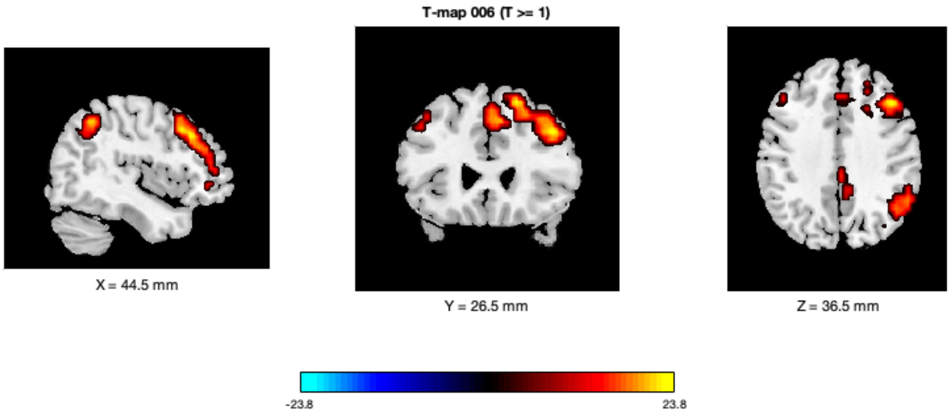

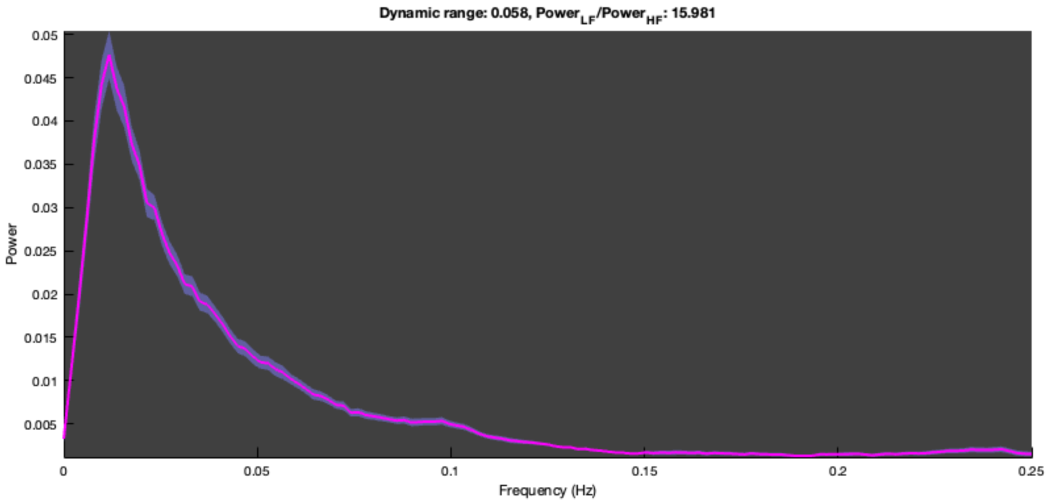

Features (Tmap 007 and Spectra)

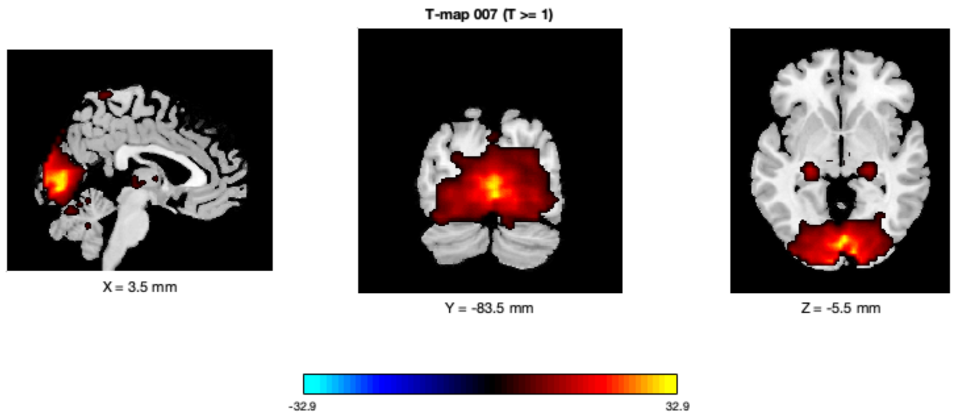

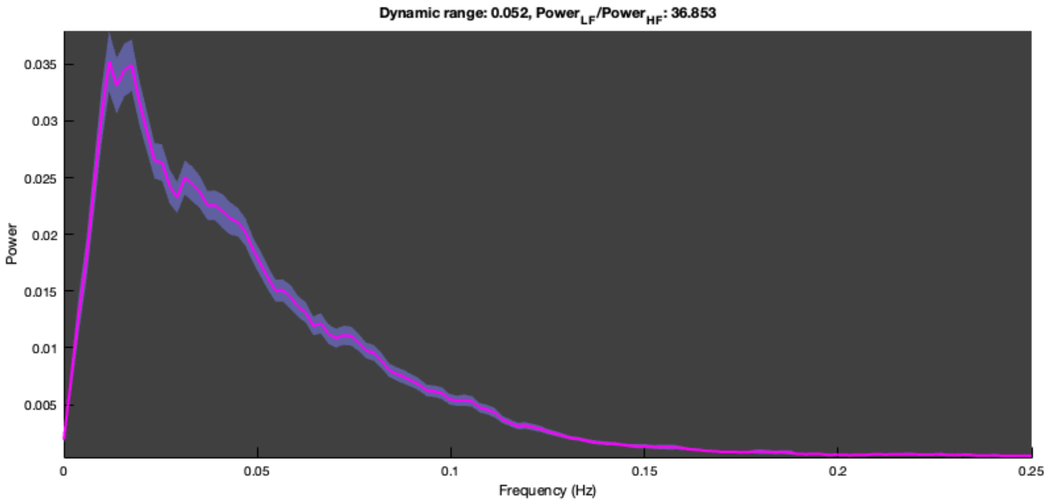

Features (Tmap 008 and Spectra)

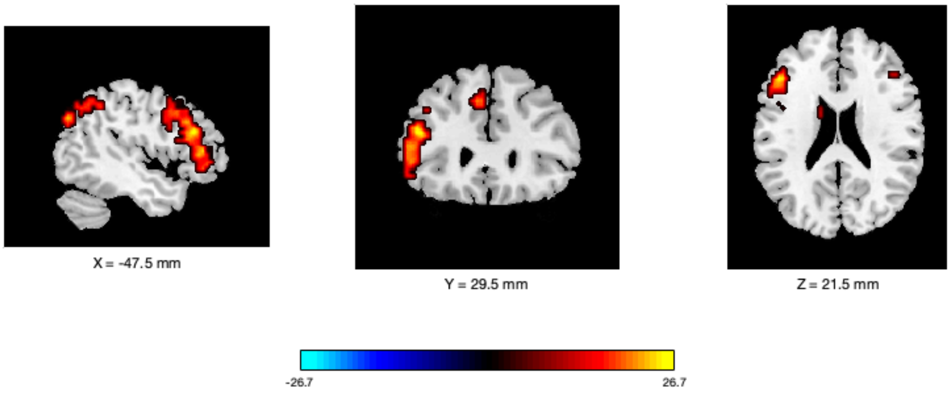

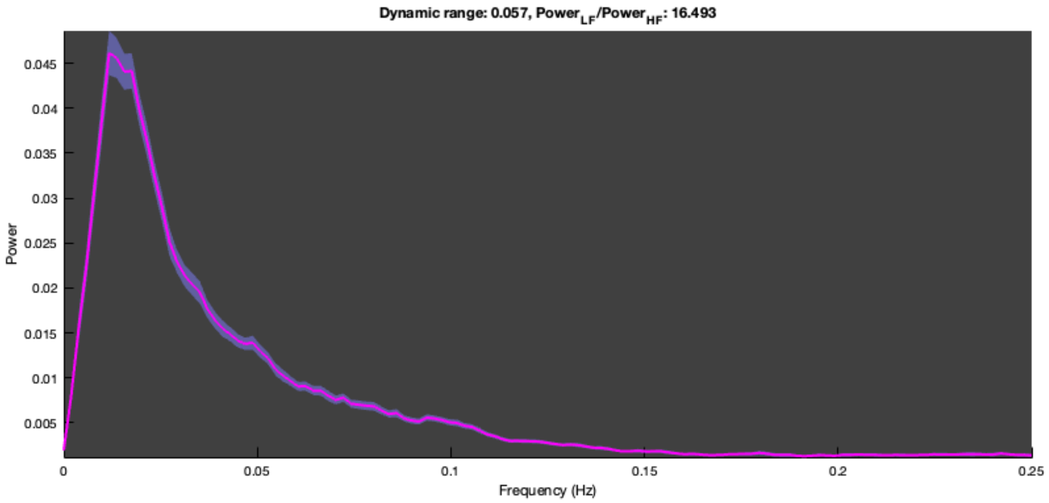

Features (Tmap 009 and Spectra)

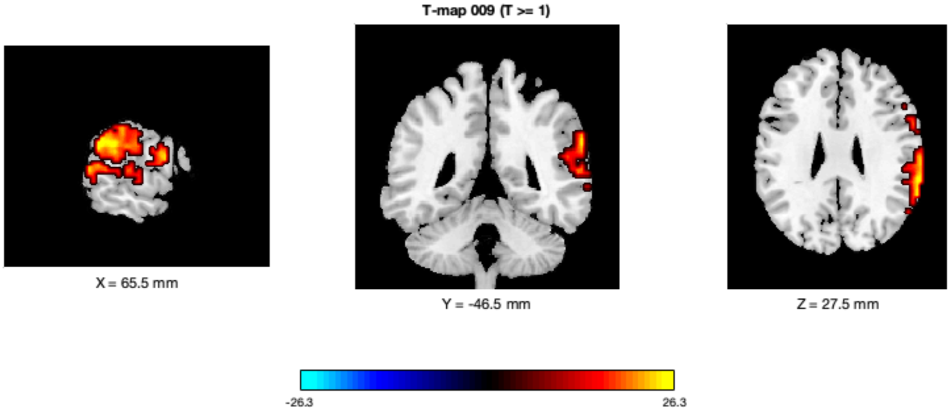

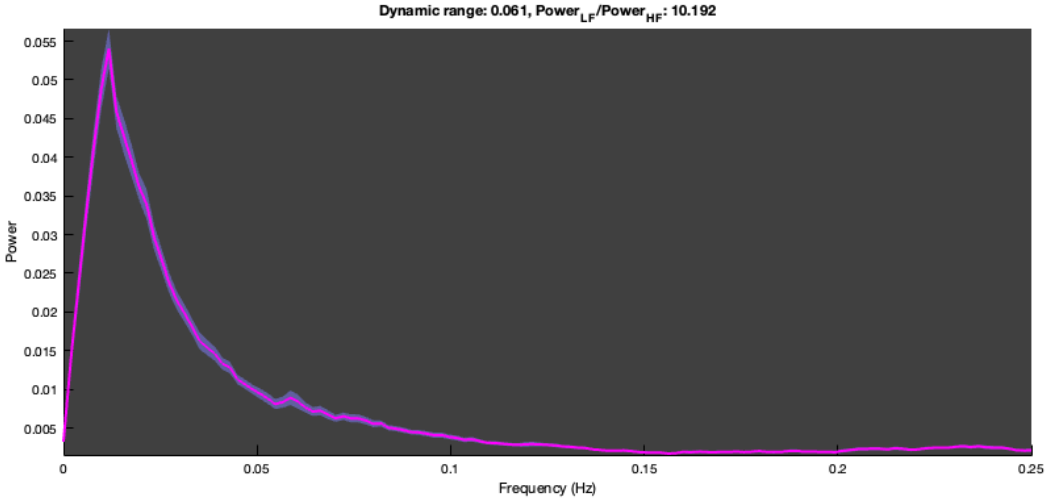

Features (Tmap 010 and Spectra)

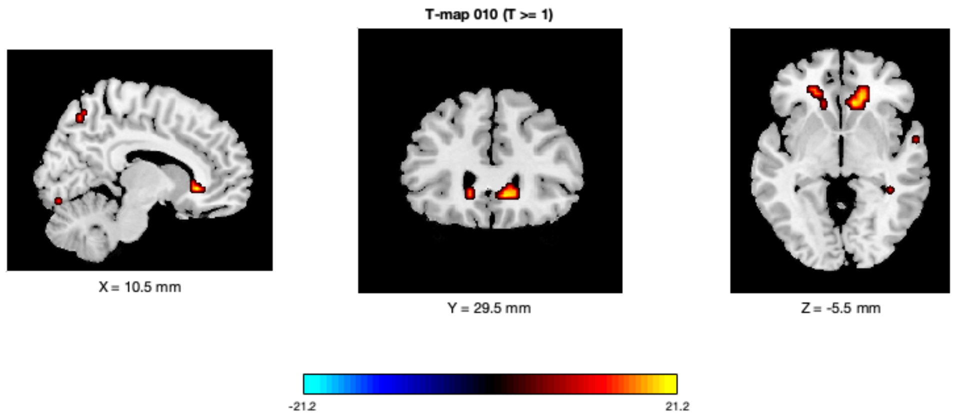

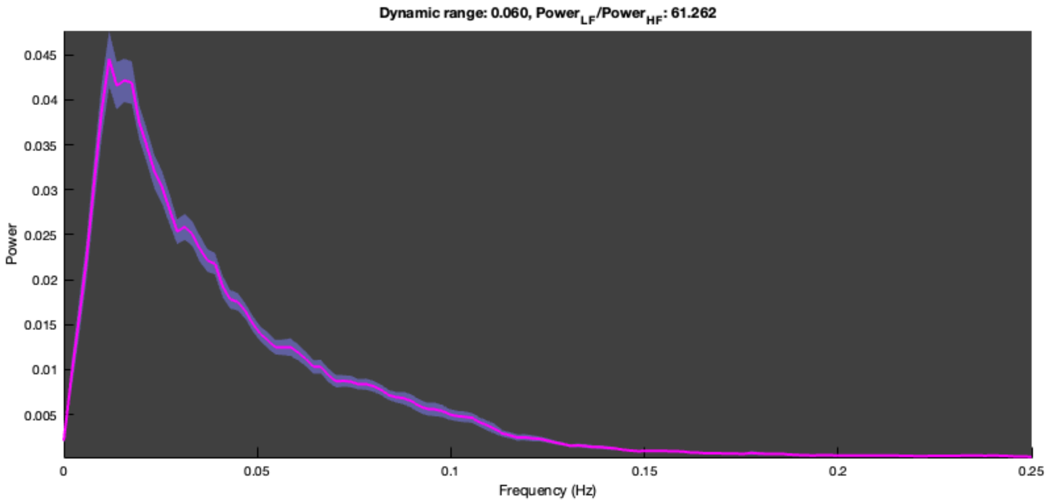

Features (Tmap 011 and Spectra)

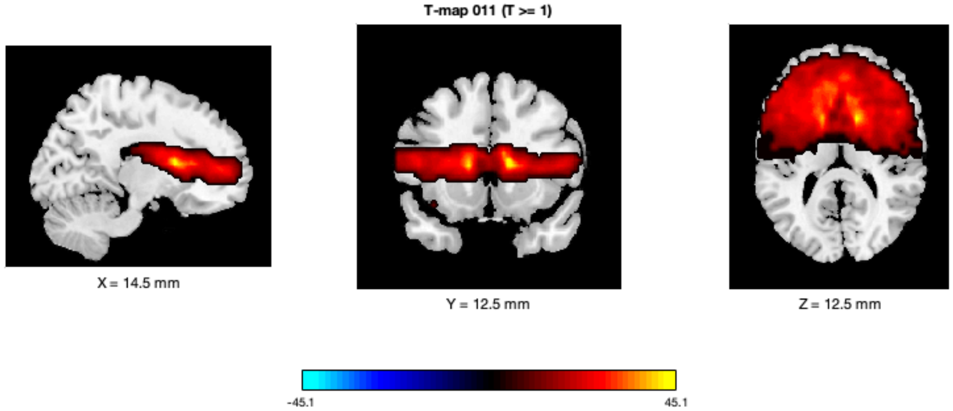

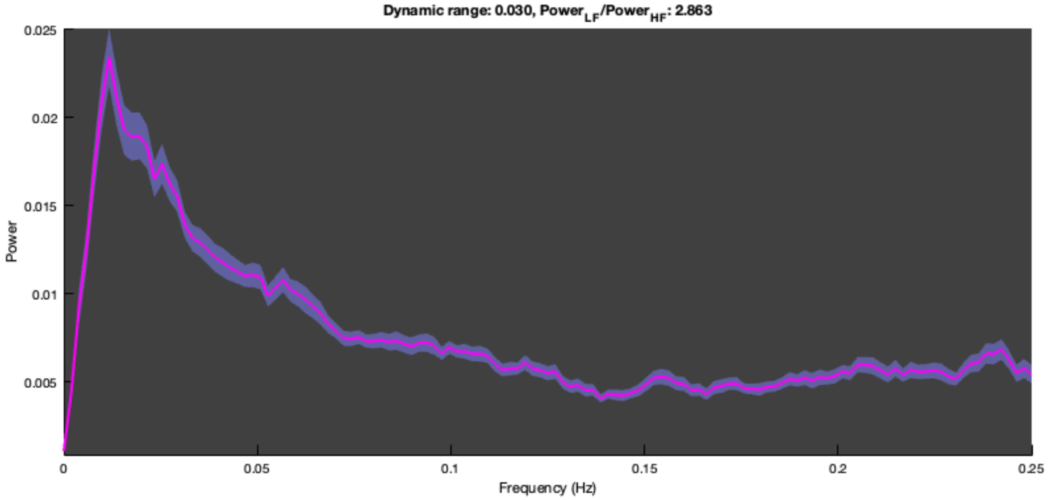

Features (Tmap 012 and Spectra)

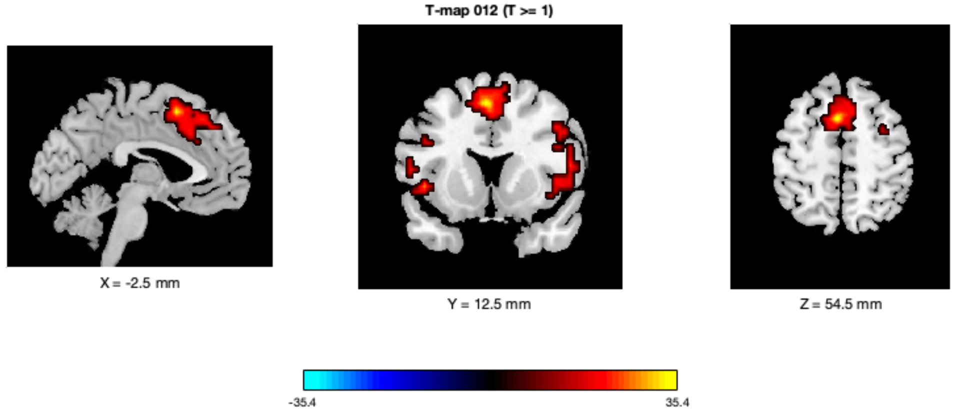

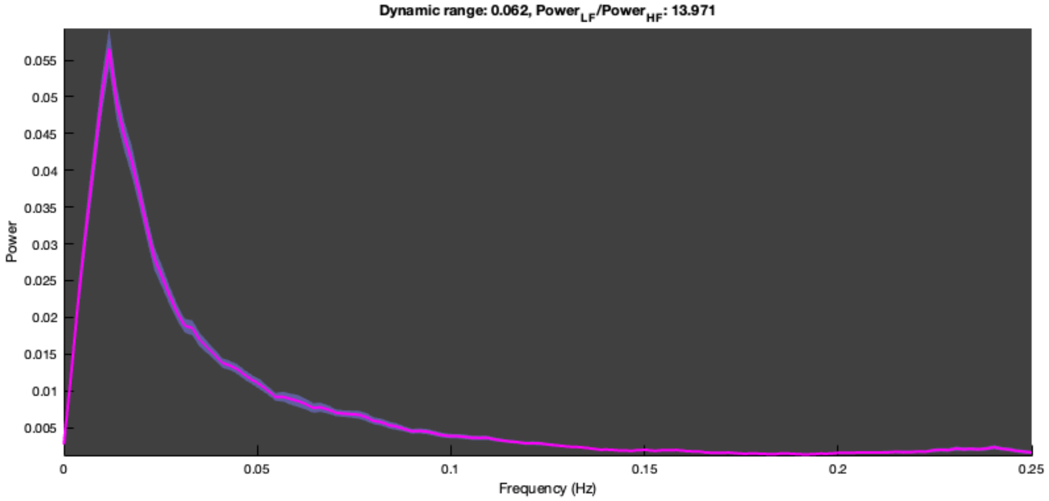

Features (Tmap 013 and Spectra)

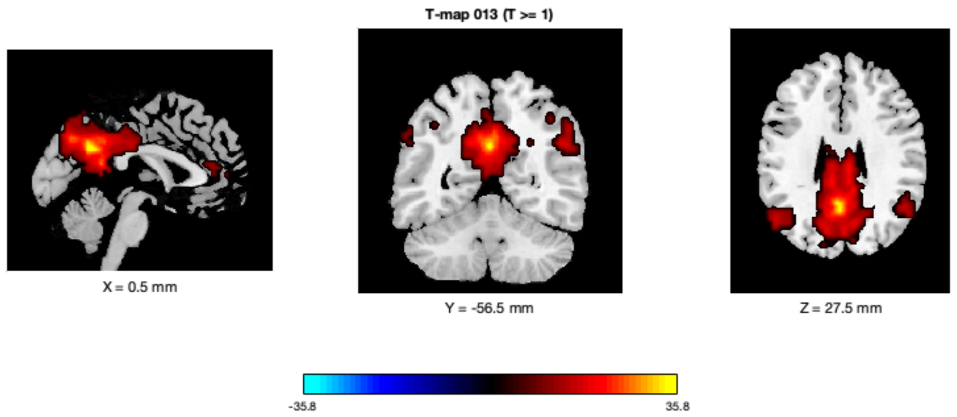

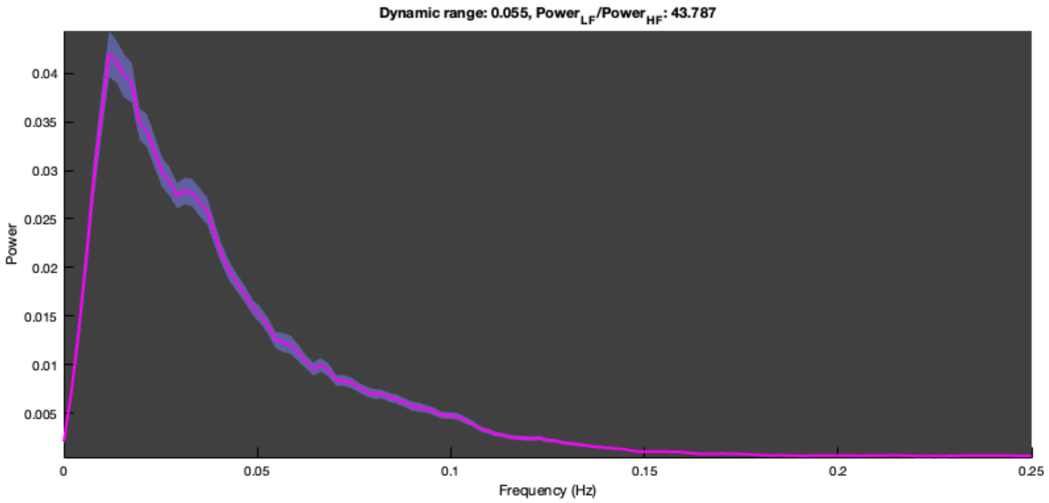

Features (Tmap 014 and Spectra)

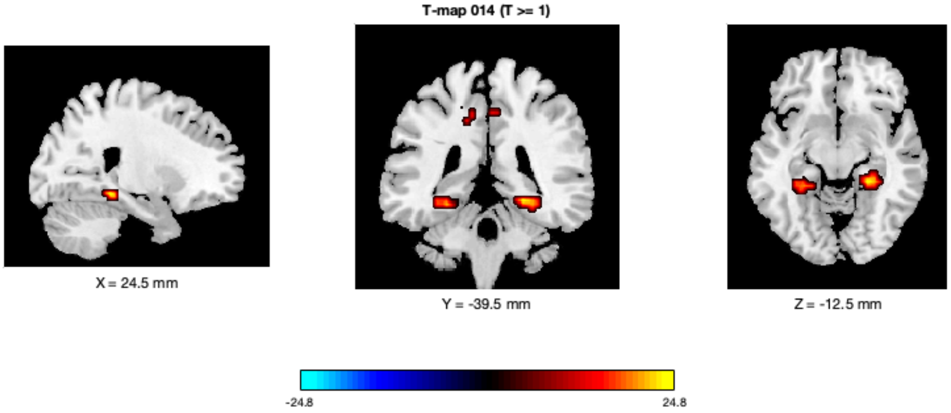

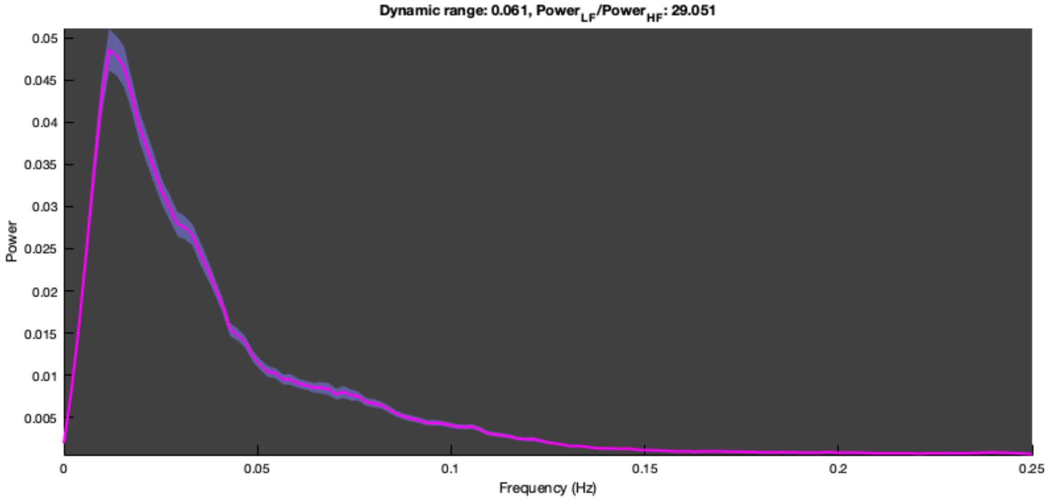

Features (Tmap 015 and Spectra)

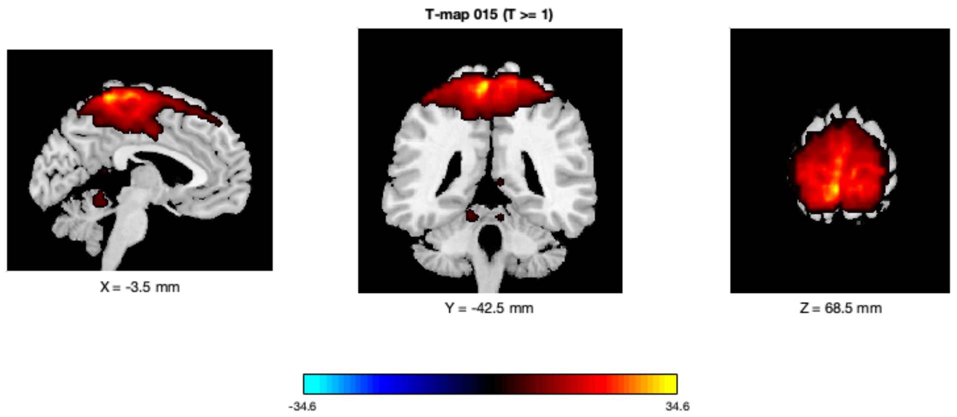

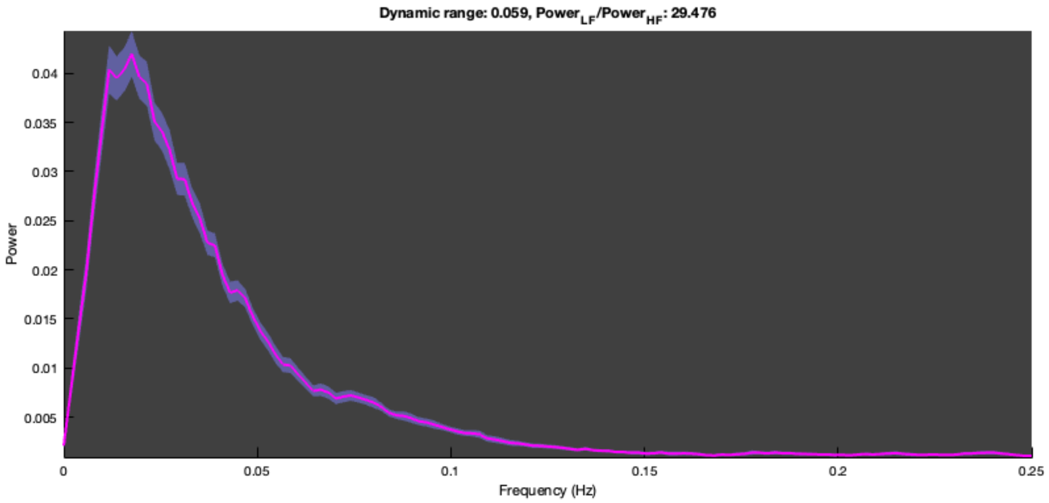

Features (Tmap 016 and Spectra)

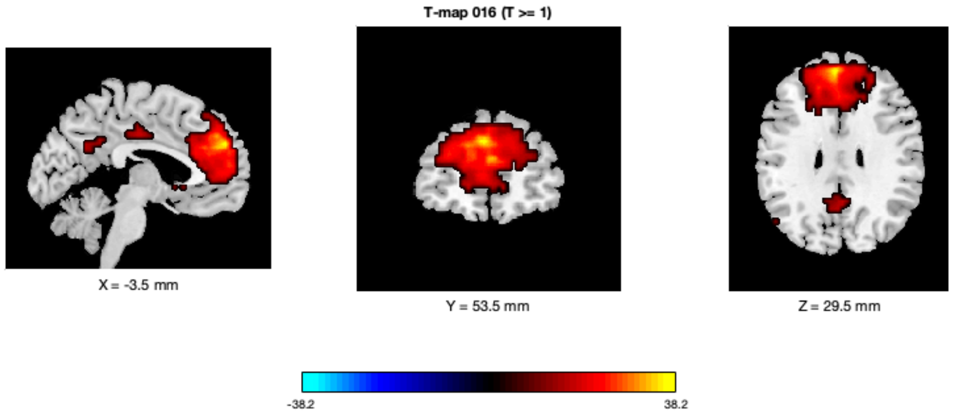

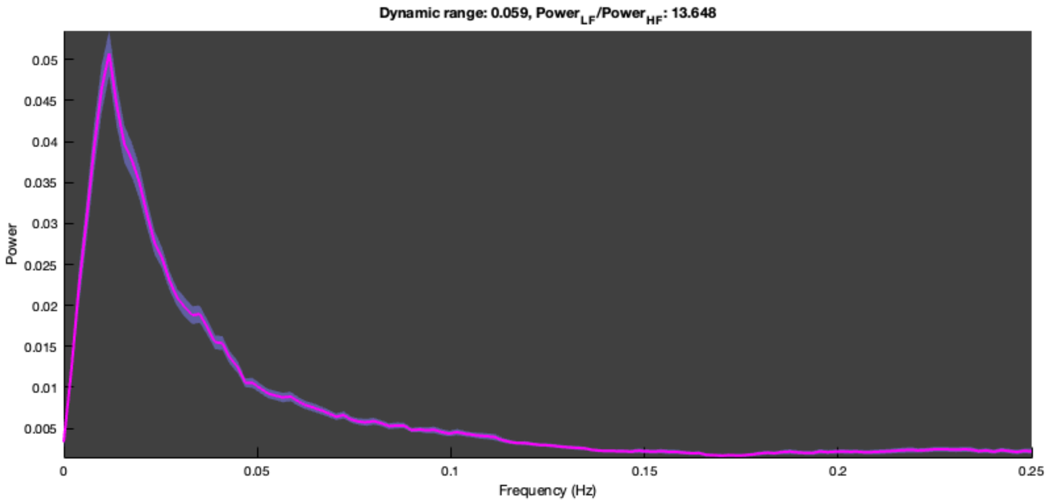

Features (Tmap 017 and Spectra)

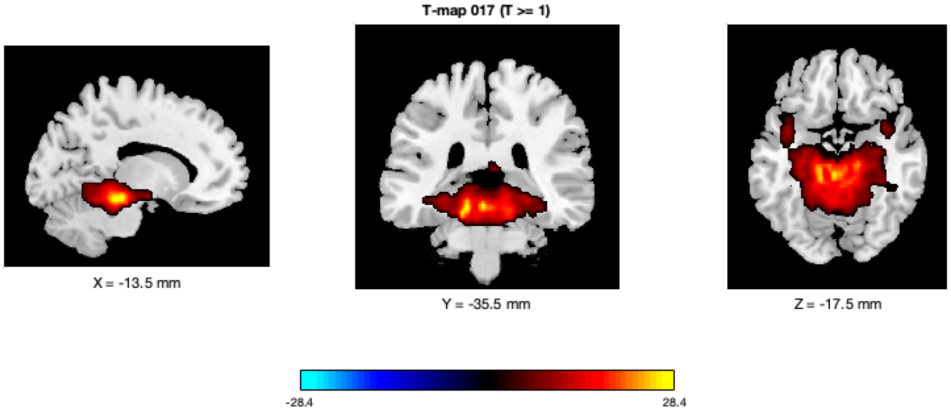

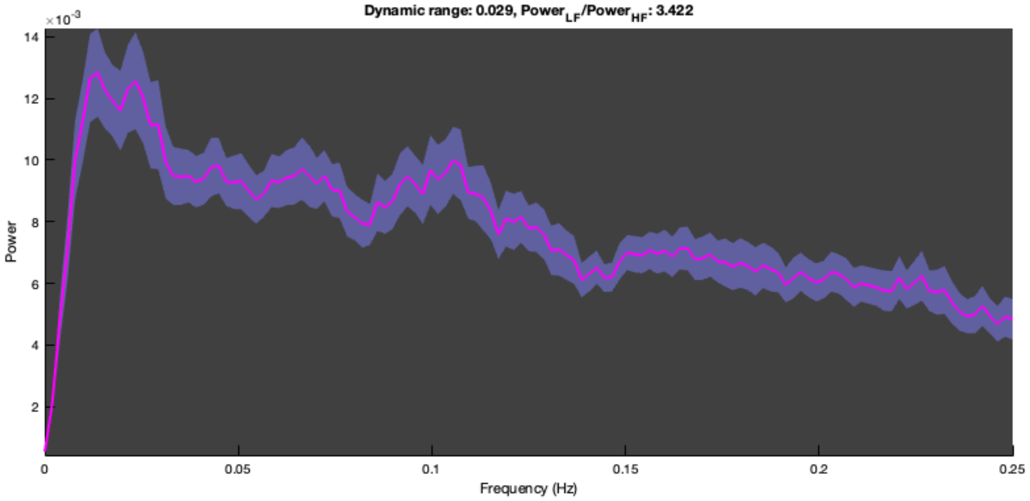

Features (Tmap 018 and Spectra)

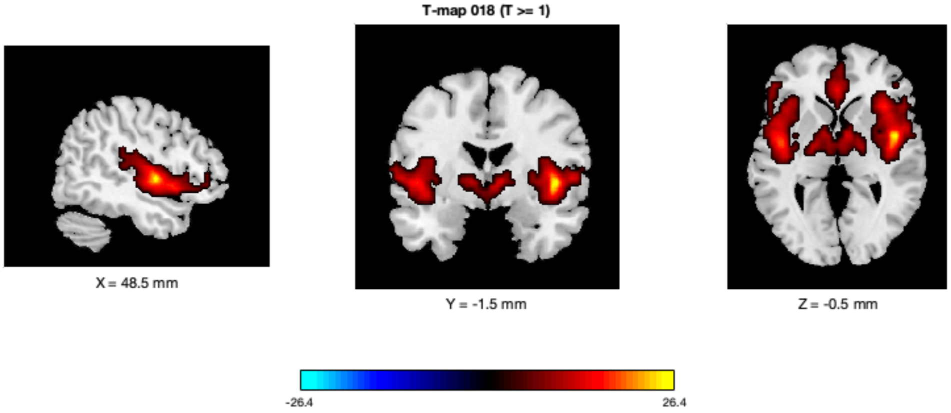

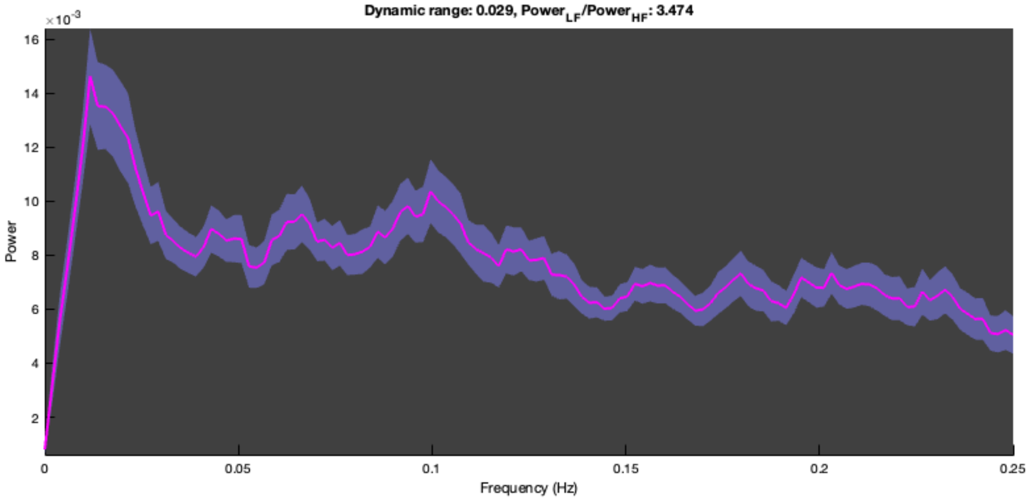

Features (Tmap 019 and Spectra)

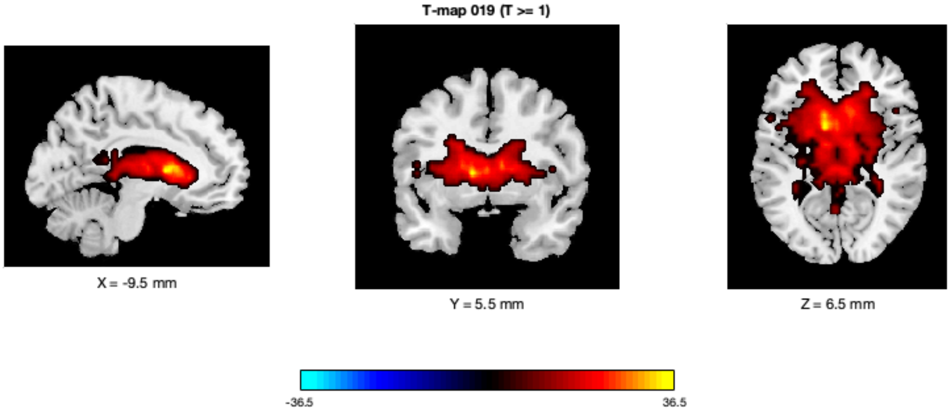

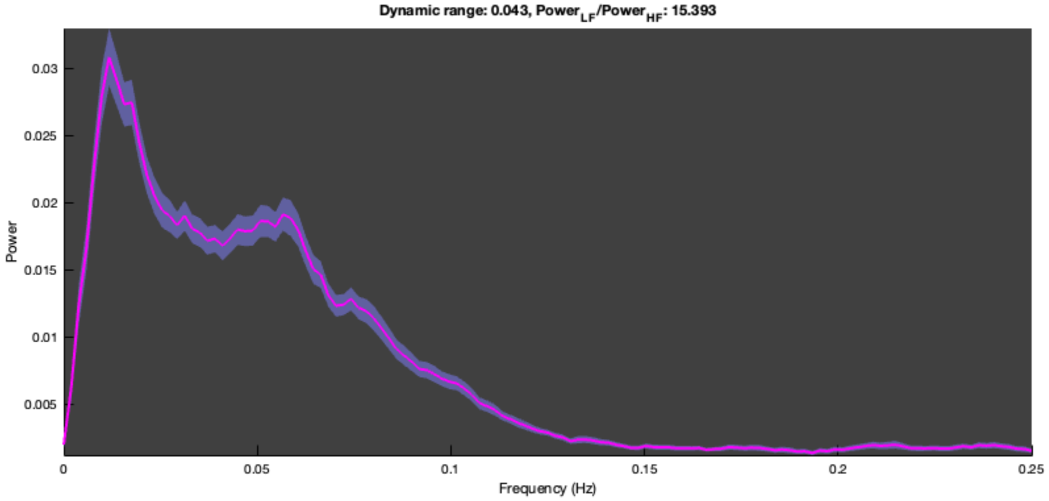

Features (Tmap 020 and Spectra)

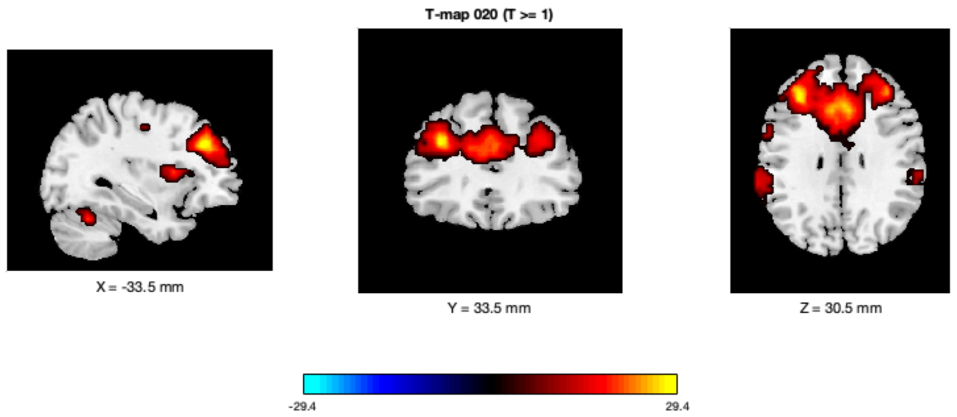

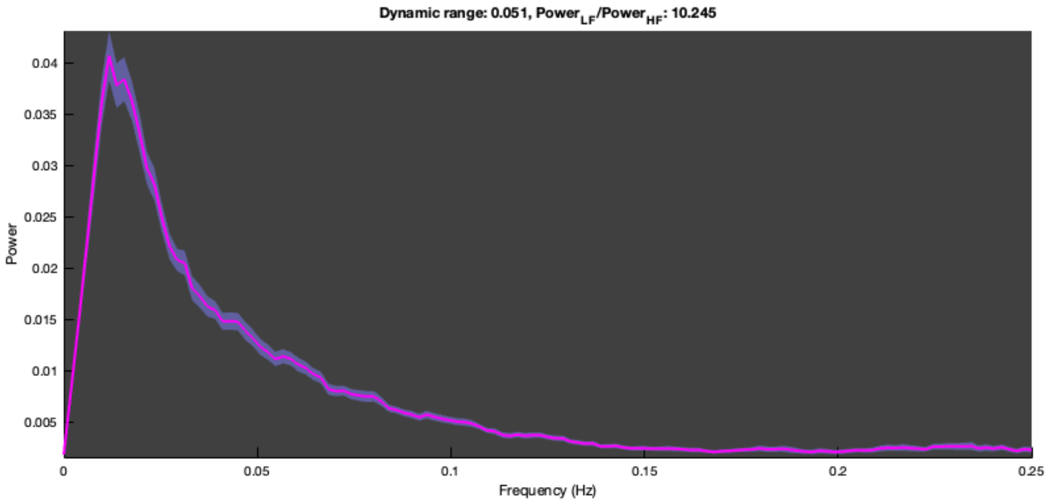

Features (Tmap 021 and Spectra)

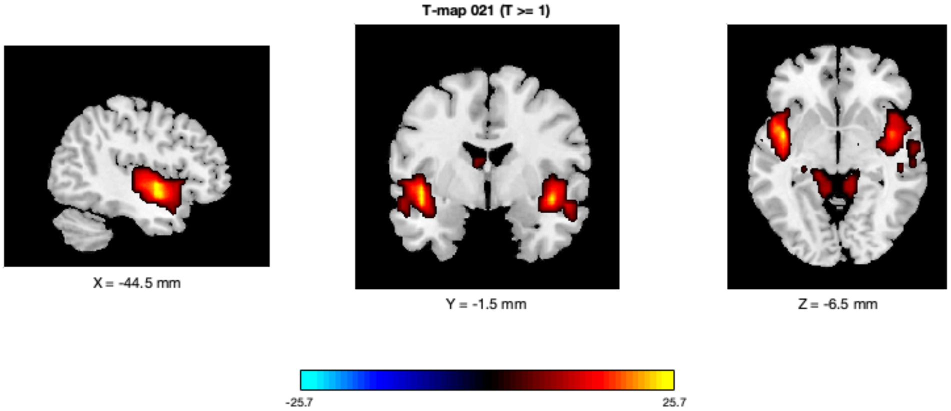

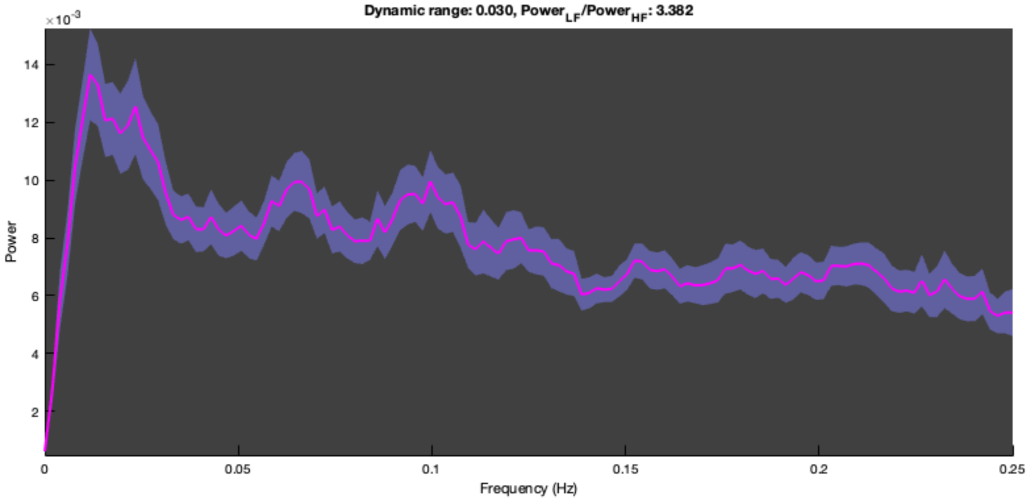

Features (Tmap 022 and Spectra)

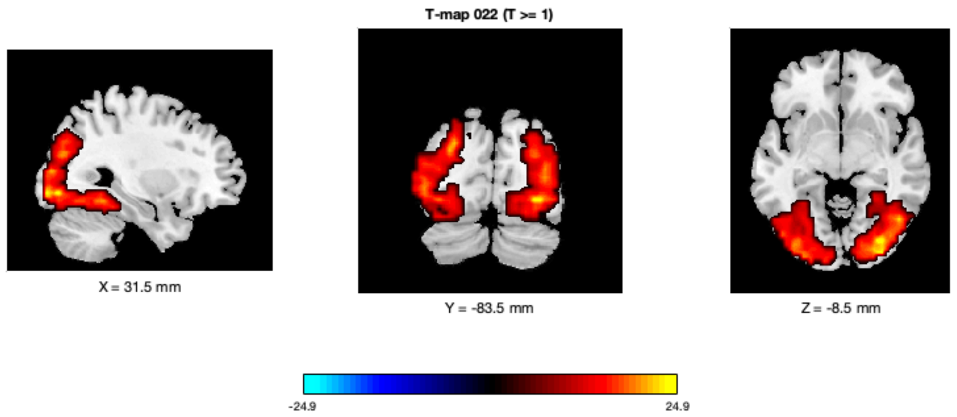

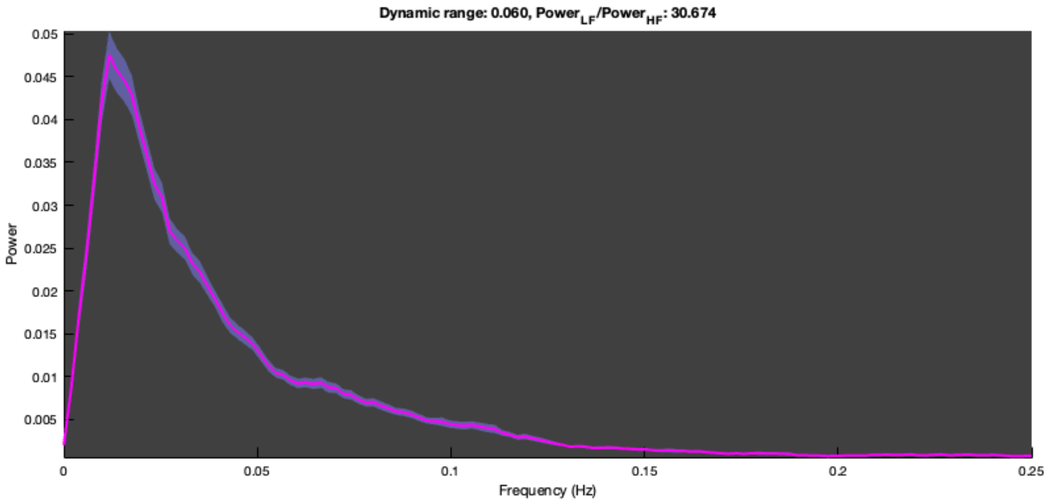

Features (Tmap 023 and Spectra)

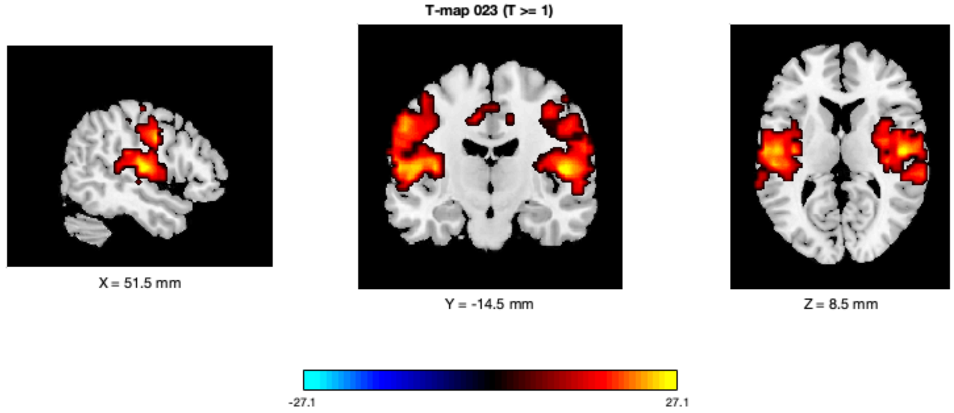

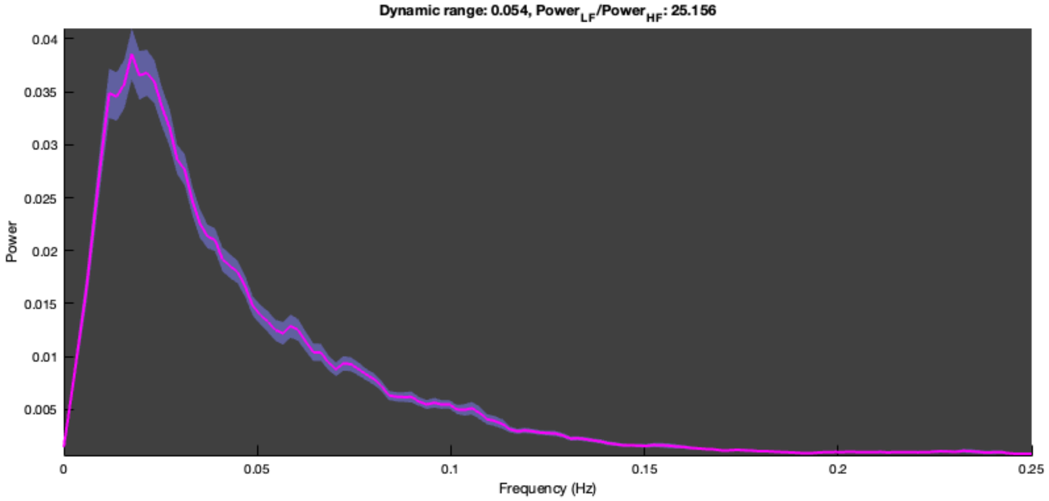

Features (Tmap 024 and Spectra)

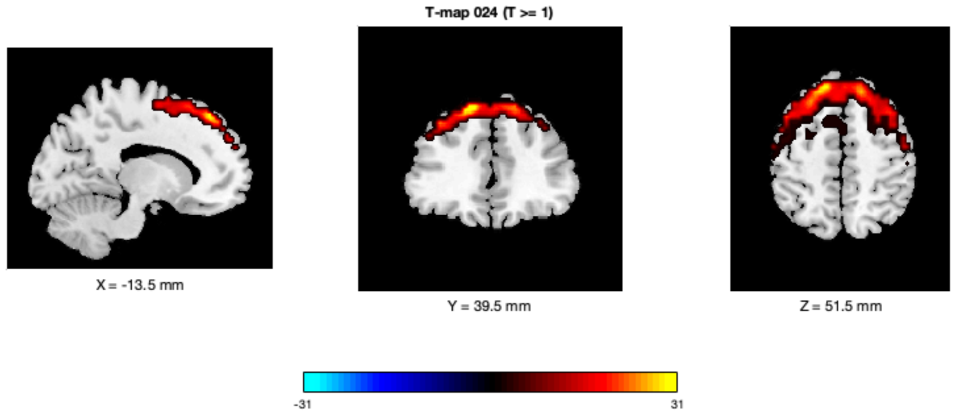

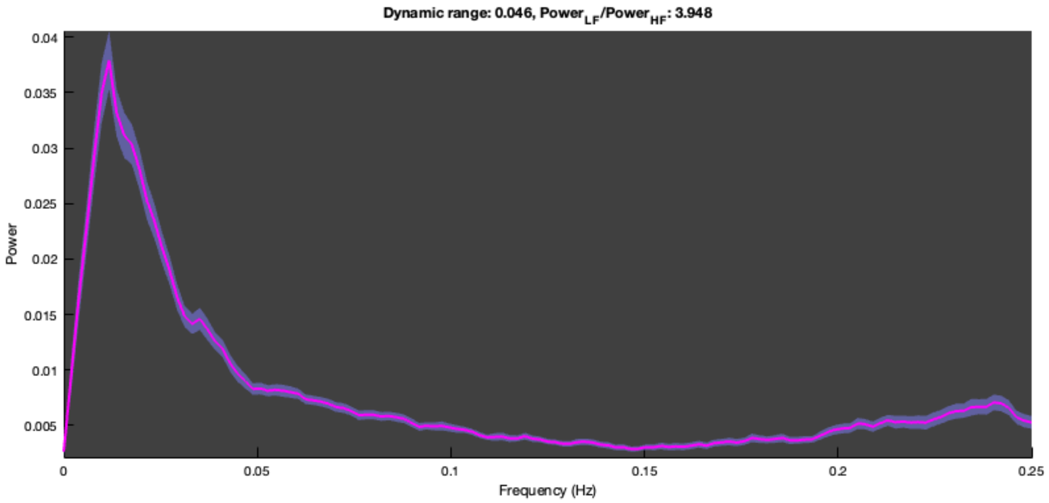

Features (Tmap 025 and Spectra)

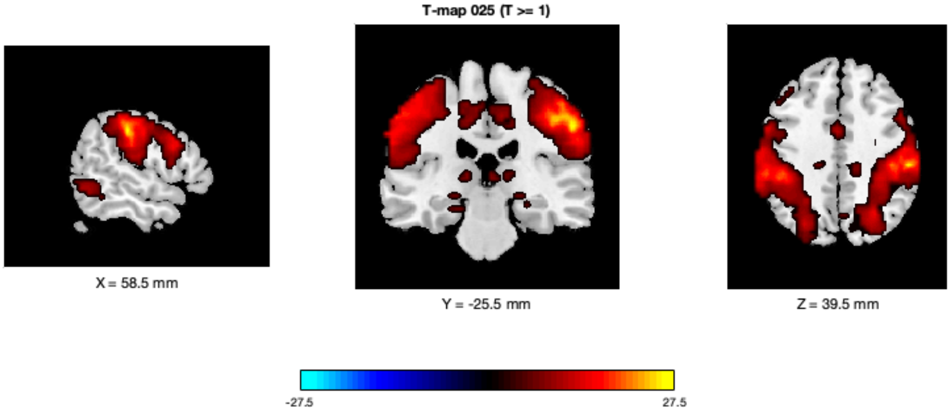

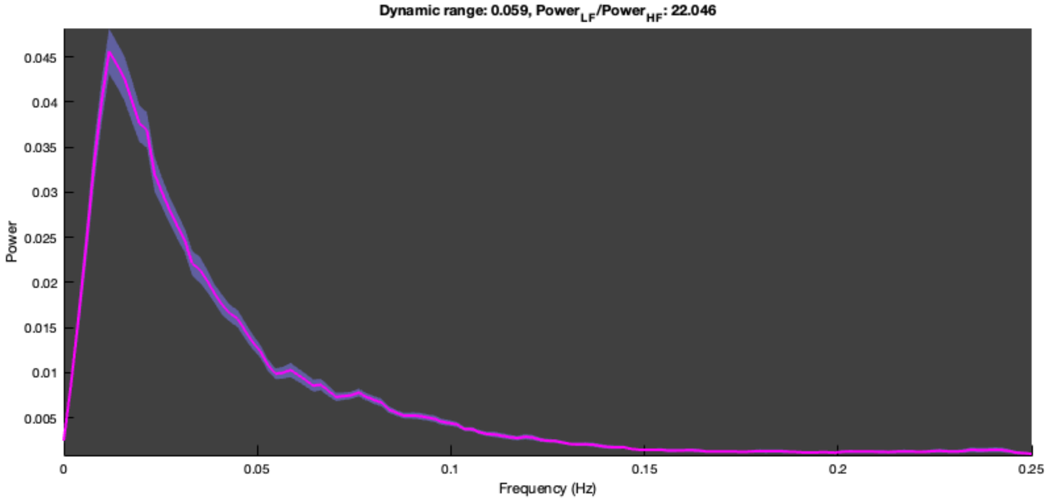

Features (Tmap 026 and Spectra)

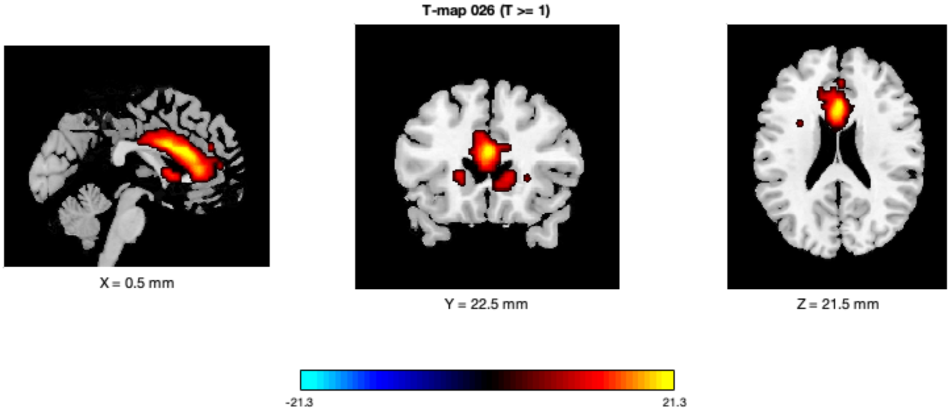

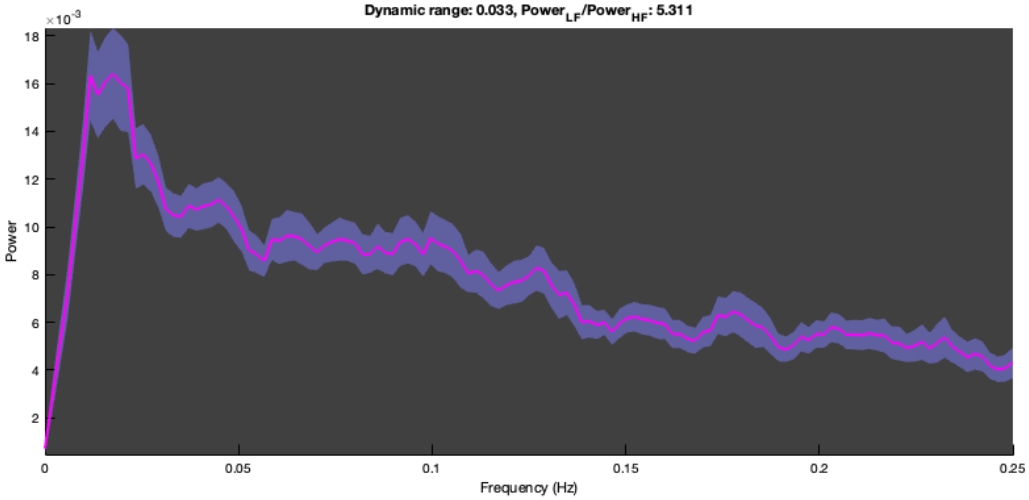

Features (Tmap 027 and Spectra)

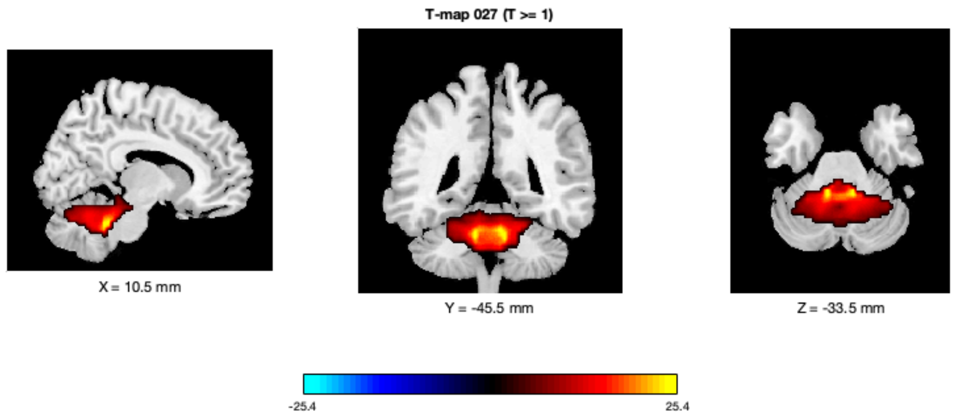

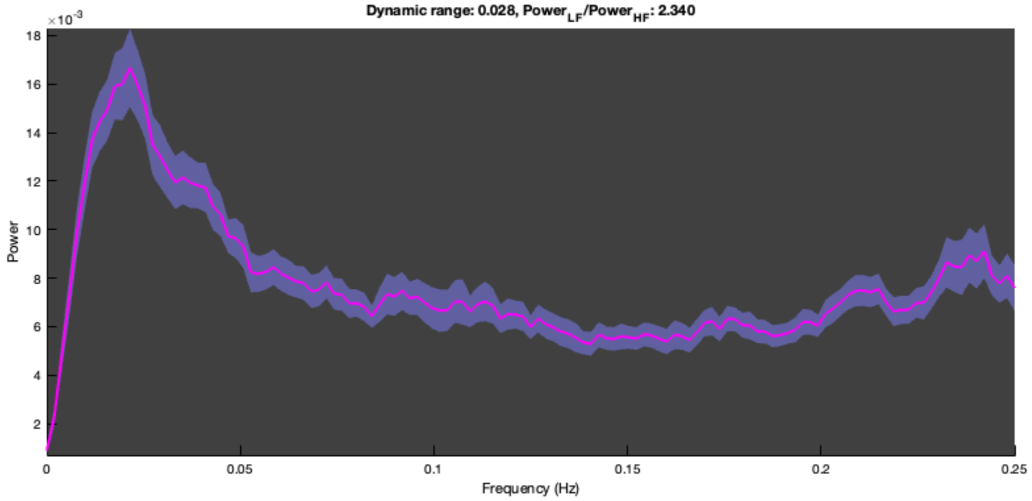

Features (Tmap 028 and Spectra)

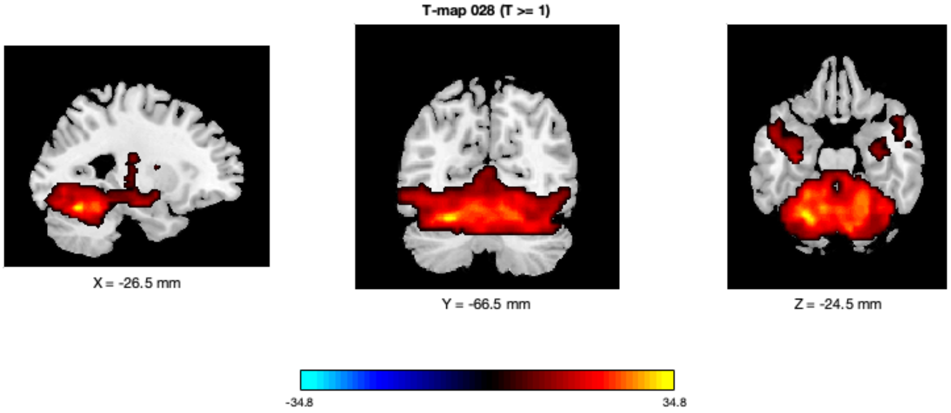

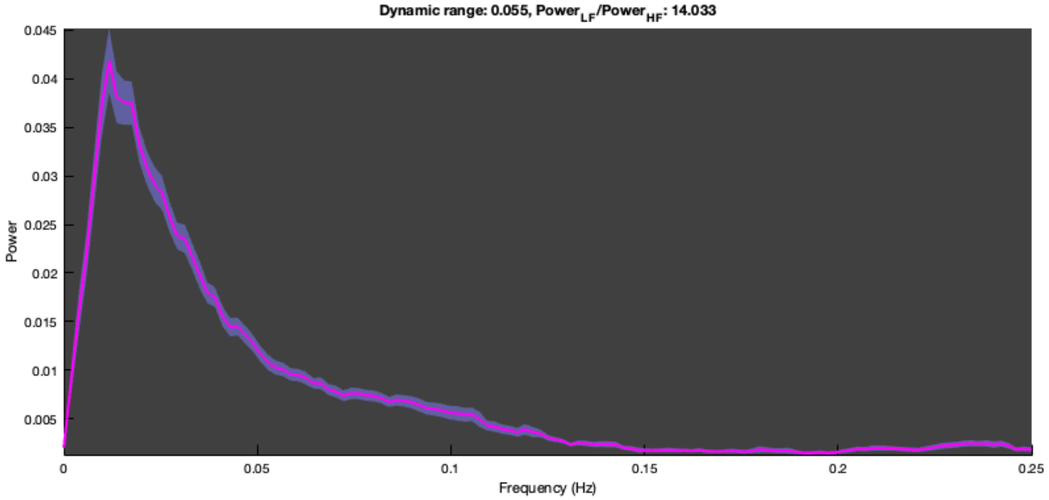

Features (Tmap 029 and Spectra)

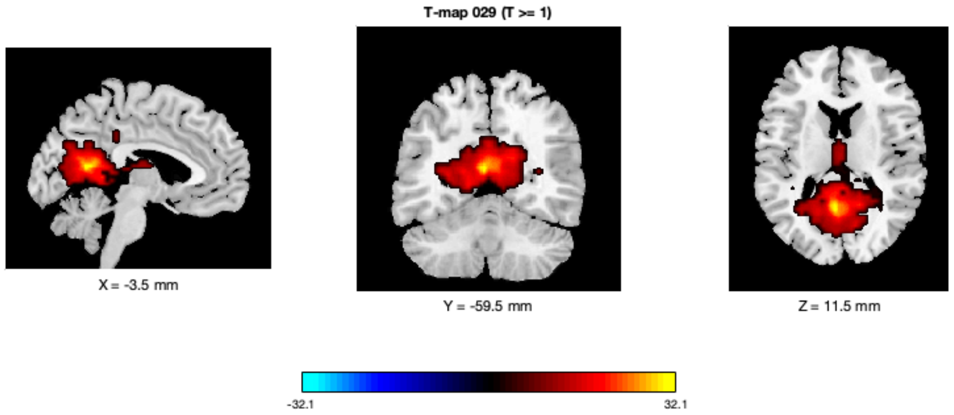

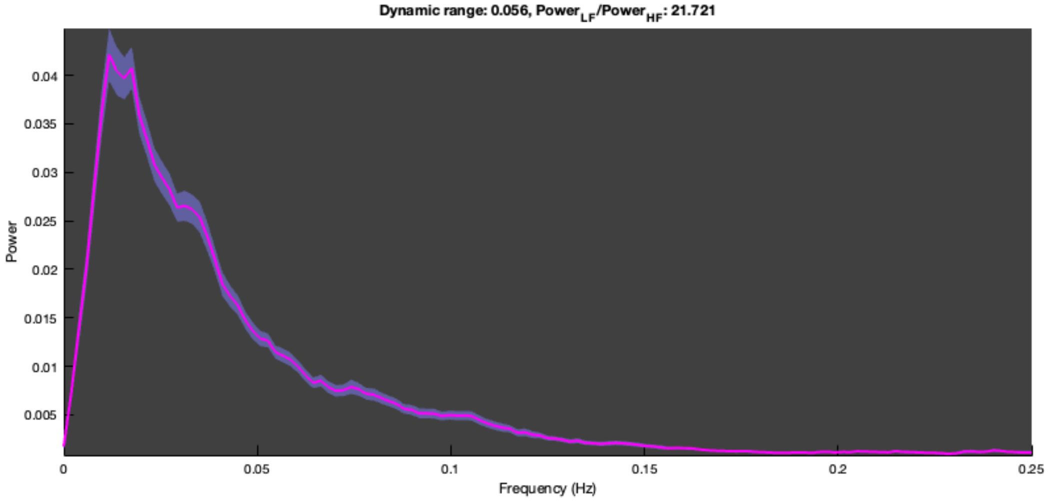

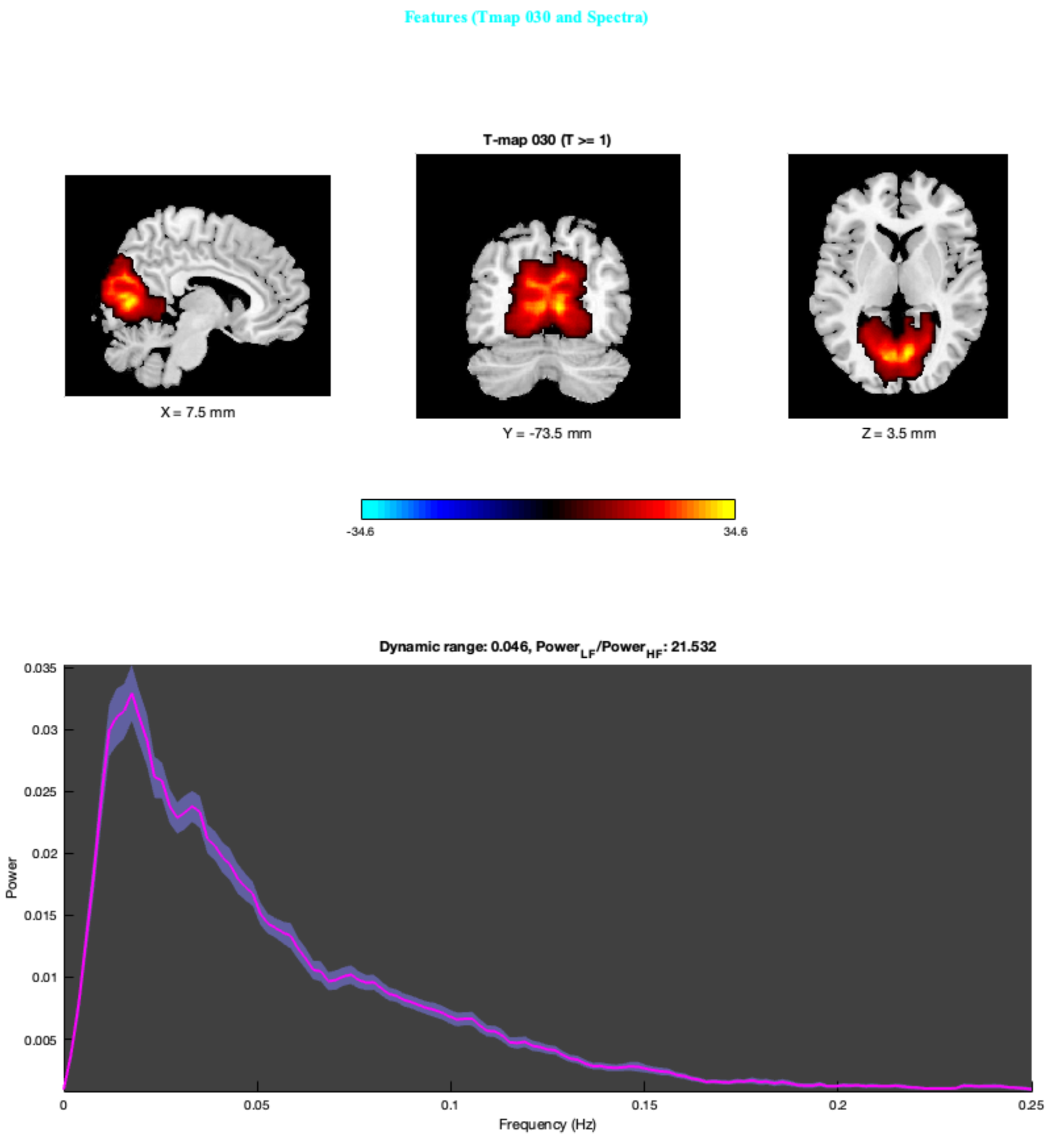

S3. References

1. Tustison NJ, Avants BB, Cook PA, Yuanjie Zheng, Egan A, Yushkevich PA, Gee JC (2010): N4ITK: Improved N3 Bias Correction. *IEEE Trans Med Imaging* 29: 1310–1320.

2. Avants BB, Epstein CL, Grossman M, Gee JC (2008): Symmetric diffeomorphic image registration with cross-correlation: Evaluating automated labeling of elderly and neurodegenerative brain. *Med Image Anal* 12: 26–41.

3. Reuter M, Rosas HD, Fischl B (2010): Highly accurate inverse consistent registration: A robust approach. *NeuroImage* 53: 1181–1196.

4. Ciric R, Rosen AF, Erus G, Cieslak M, Adebimpe A, Cook PA, *et al.* (2018): Mitigating head motion artifact in functional connectivity MRI. *Nat Protoc* 13: 2801–2826.

5. Fonov V, Evans A, McKinstry R, Almli C, Collins D (2009): Unbiased nonlinear average age-appropriate brain templates from birth to adulthood. *NeuroImage* 47: S102.

6. Jenkinson M, Bannister P, Brady M, Smith S (2002): Improved Optimization for the Robust and Accurate Linear Registration and Motion Correction of Brain Images. *NeuroImage* 17: 825–841.

7. Jenkinson M, Smith S (2001): A global optimisation method for robust affine registration of brain images. *Med Image Anal* 5: 143–156.

8. Greve DN, Fischl B (2009): Accurate and robust brain image alignment using boundary-based registration. *NeuroImage* 48: 63–72.

9. Power JD, Mitra A, Laumann TO, Snyder AZ, Schlaggar BL, Petersen SE (2014): Methods to detect, characterize, and remove motion artifact in resting state fMRI. *NeuroImage* 84: 320–341.

10. Abraham A, Pedregosa F, Eickenberg M, Gervais P, Mueller A, Kossaifi J, *et al.* (2014): Machine learning for neuroimaging with scikit-learn. *Front Neuroinformatics* 8. https://doi.org/10.3389/fninf.2014.00014
